# Lipopolysaccharide Preconditioning Restricts Microglial Overactivation and Alleviates Inflammation-Induced Depressive-like Behavior in Mice

**DOI:** 10.3390/brainsci13040549

**Published:** 2023-03-25

**Authors:** Haiping Yu, Junli Kan, Mingming Tang, Yanbing Zhu, Baoyang Hu

**Affiliations:** 1 State Key Laboratory of Stem Cell and Reproductive Biology, Institute of Zoology, Chinese Academy of Sciences (CAS), Beijing 100101, China; 2 Institute for Stem Cell and Regeneration, Chinese Academy of Sciences, Beijing 100101, China; 3 University of Chinese Academy of Sciences, Beijing 100049, China; 4 Beijing Institute for Stem Cell and Regenerative Medicine, Beijing 100101, China; 5 Savaid Medical School, University of Chinese Academy of Sciences, Beijing 101408, China; 6 Beijing Clinical Research Institute, Beijing Friendship Hospital, Capital Medical University, Beijing 100050, China; 7 National Stem Cell Resource Center, Institute of Zoology (CAS), Beijing 100190, China

**Keywords:** microglia, neuroinflammation, depression, mouse model of inflammation-induced depression, LPS, G-protein-coupled receptor 84 (GPR84)

## Abstract

Overactive microglia and severe neuroinflammation play crucial roles in the development of major depressive disorder. Preconditioning with lipopolysaccharide (LPS) provides protection against severe neuroinflammation. However, administering high doses of LPS to mice triggers depressive symptoms. Therefore, the optimal dose of LPS preconditioning needs to be determined by further experiments. LPS preconditioning is an effective agent in anti-inflammation and neuroprotection, but the mechanism by which LPS preconditioning acts in depression remain unclear. This study finds that the anti-inflammation mechanism of low-dose LPS preconditioning is mainly dependent on G-protein-coupled receptor 84 (GPR84). We use low-dose LPS for preconditioning and re-challenged mice or BV2 microglia with high-dose LPS. In addition, RNA-seq is used to explore underlying changes with LPS preconditioning. Low-dose LPS preconditioning reduces the expression of pro-inflammatory mediators and inhibits microglial activation, as well as suppresses the depressive-like behavior when the mice are re-challenged with high-dose LPS. Further investigation reveals that the tolerance-like response in microglia is dependent on the GPR84. Here, we show that low-dose LPS preconditioning can exert anti-inflammation effects and alleviates inflammation-induced depressive-like behavior in mice. As a potential therapeutic target for depression, LPS preconditioning needs to be given further attention regarding its effectiveness and safety.

## 1. Introduction

Major depressive disorder (MDD) is a common and serious mental disorder that currently affects millions of people worldwide [1,2]. Increasing evidence has suggested a critical link between depression and innate immunity, including the dual detrimental and protective roles of activated microglia in depression [3,4].

Microglia are resident macrophages of the central nervous system (CNS) and play an important role in health and disease [5,6]. Under physiological conditions, microglia are highly ramified and patrol the CNS, dynamically monitoring changes in an effort to maintain homeostasis of the microenvironment [7,8,9]. Microglia are activated in many neurological diseases, including MDD, and rapidly adapt their phenotype and functions in response to the brain milieu. Once an injury has been resolved or a stimulus has been removed, microglia return to their resting state. However, if microglia are overactivated, they release massive amounts of proinflammatory cytokines, which can harm neurons and even exacerbate neurological disorders [10,11]. For example, administration of lipopolysaccharide (LPS) (0.83 mg/kg or 1 mg/kg), a common stimulant of the innate immune system, has been shown to induce hyperactivation of microglia, severe neuroinflammation and depressive symptoms in mice [12,13]. In addition, patients treated with IFN-α may develop depressive symptoms due to neuroinflammation [14]. Epidemiological findings suggest that MDD patients have elevated levels of proinflammatory mediators in plasma [15]. Overactivated microglia are commonly found in depressive patients’ brains, according to clinical studies [16,17]. There is also evidence that chronic inflammation may increase the risk of MDD [18]. These findings further suggest that depression is highly associated with neuroinflammation. Clinically used antidepressants can inhibit microglial activation and reduce proinflammatory cytokine production [19,20]. Additionally, minocycline, a microglial inhibitor, ameliorates depressive-like symptoms induced by LPS or IFN-α [21,22]. Therefore, modulating microglia to reduce neuroinflammation is considered a promising strategy for the treatment of depression.

Microglia can be polarized toward two main phenotypes: the proinflammatory M1 (classically activated) and anti-inflammatory M2 (sometimes subdivided into alternatively activated and acquired deactivation) phenotypes [23]. Studies have shown that M1 microglia promote depression in mice by releasing a large number of proinflammatory cytokines [24,25]. To prevent the exacerbation of the inflammatory response in the brain, microglia need to be strictly regulated. Exposure of innate immune cells to LPS leads to a hyporeactive state, resulting in reduced production of inflammatory cytokines in response to subsequent LPS challenge, a phenomenon known as endotoxin tolerance [26]. The main effector cells for endotoxin tolerance are monocytes/macrophages. Although endotoxin tolerance in the peripheral has been well studied, little is known about endotoxin tolerance in the brain. Recently, endotoxin tolerance has been observed in microglia. For example, LPS treatment induces an anti-inflammatory phenotype in BV2 microglia, characterized by reduced expression of proinflammatory cytokines and chemokines [27]. LPS preconditioning (0.25 mg/kg) suppresses inflammation in the brain for at least 32 weeks [28]. In addition, LPS preconditioning exerts a neuroprotective effect in animal models of ischemia (retinal ischemia and middle cerebral artery occlusion) [29,30]. These suggest that LPS preconditioning may be a novel strategy for treating depression. However, intraperitoneal injection of 0.83 mg/kg or 1 mg/kg of LPS induces depressive symptoms in mice. Therefore, further work is needed to identify the threshold dose of LPS and test whether it exerts antidepressant effects.

The gray matter density in the hippocampus, amygdala, and prefrontal cortex (PFC) of the brain is significantly decreased in depressed patients compared to healthy individuals. PFC is a key brain region in depression, and its dysfunction leads to impairment of aversive learning and emotional memory circuits [31]. Alterations in the molecular regulation of higher-order neural circuits and neuropathology may lead to PFC dysfunction [32,33]. The hippocampus is an important brain region for memory preservation and recall of memories, and the hippocampus is more biased toward negative emotional memories and recall in depression. Depression is a state of stress that disrupts the normal function of the hippocampus, not only exhibiting abnormal activity, but in severe cases, leading to the atrophy of hippocampal volume [34,35,36]. This atrophy may be due to chronic stress that over-activates microglia, which in turn leads to neuronal death. Thus, we have focused on microglial changes in the hippocampus.

An increasing number of studies have focused on the role of the tryptophan-kynurenine (Trp-Kyn) metabolic system, not only in the pathogenesis of diseases, but also as a potential biomarker of environmental health [37,38]. The Trp–Kyn metabolic system refers to a group of endogenous bioactive metabolites produced by the essential amino acid Trp via the Kyn metabolic pathway. They interact with the immune system and are involved in the tolerance shift of chronic low-grade inflammation [39]. The proportion of Kyn and Trp was significantly higher in the blood of depressed patients, and was positively correlated with anxiety and cognitive impairment in depressed patients [40]. Indoleamine-2,3-dioxygenase 1 (IDO1) is a Trp-degrading enzyme, of which the activation is related to the occurrence of depressive behavior. Activated microglia can metabolize Trp to canine Kyn by expressing IDO, this depletes Trp content and decreases 5-HT synthesis [41]. IDO1 activation in the brain is sufficient to cause depressive-like behaviors in mice [42]. In addition, inhibiting IDO1 ameliorates depressive-like behaviors in chronic unpredictable mild stress (CUMS) mice [43]. The levels of IDO1 are mainly increased in microglia in the brains of mice with poststroke depression (PSD) [44]. Therefore, increased expression of IDO1 is an important feature of depression. M1/M2 polarization of microglia is related to the ERK signal transduction. LPS treatment increases ERK phosphorylation, while inhibition of ERK phosphorylation reduces the M1 polarization of microglia and the microglia-mediated inflammatory response [45]. This suggests that ERK signaling may be important for microglial tolerance.

In this study, we sought to identify the threshold dose of LPS and test whether it exerts antidepressant effects in mice. Interestingly, we observed that 10 µg/kg LPS preconditioning inhibited microglia-mediated neuroinflammation and alleviated depressive-like behavior in a mouse model of inflammation-induced depression.

## 2. Materials and Methods

### 2.1. Animals

Male C57BL/6J mice of 8 weeks (22–24 g) were obtained from SiPeiFu Biotechnology Co., Ltd (Beijing, China). Mice were group-housed with a maximum of 5 animals per cage and had free access to food and water at room temperature, with a constant 12:12 h light–dark cycle at the Experiment Animal Center of Institute of Zoology, Chinese Academy of Sciences. All animal experiments were carried out in accordance with the guidelines approved by the Institutional Animal Care and Use Committee of the Institute of Zoology, Chinese Academy of Sciences (IOZ-IACUC-2021-105).

### 2.2. Cell Culture

The murine BV2 microglial cell lines were cultured in Dulbecco’s modified eagle medium (DMEM; Gibco, Carlsbad, CA, USA), supplemented with 10% (*v*/*v*) fetal bovine serum (FBS; Gibco) and penicillin/streptomycin (100 units and 100 μg/mL, Gibco), and incubated at 37 °C in a humidified incubator with 5% CO_2_.

The BV2 cell line was an immortalized cell line obtained by Blasi et al. in 1990, by infecting primary-cultured mouse microglia with the retrovirus J2 carrying the oncogene *v- raf*/ *v- myc* [46]. This cell line was not only highly purified, but also had the morphological, phenotypic and functional characteristics of primary-cultured microglia, and was relatively easy to be cultured.

### 2.3. Drugs

LPS (derived from *E. coli* serotype 0111: B4, L-2880, Sigma, St Louis, MO, USA) was dissolved in DPBS or physiological saline (for the in vivo experiment). Zymosan A (Zym; S11175, Yuanye, China) and β-glucose (β-glu; S24487, Yuanye, China) were dissolved in DPBS. LPS, Zym, and β-glu were all used as inflammation inducers.

### 2.4. Experimental Design

We performed three animal experiments and three cellular experiments with the aim of demonstrating that low-dose LPS preconditioning did not induce depressive-like behavior in mice, but exerted anti-inflammatory and antidepressant effects, and investigated the mechanisms involved.

#### 2.4.1. In Vivo Experiments

1. Experiment 1. Repeated challenge with 5 mg/kg LPS in mice

Mice were treated with high doses of LPS (5 mg/kg, twice, 6 days apart). Behavioral tests were carried out 3 h after the second LPS injection. Mice were divided into 3 groups: the Ctrl group (saline–saline), LPS group (saline–5 mg/kg LPS), and RL group (5 mg/kg LPS–5 mg/kg LPS). Each group had 10 mice, aged 8–10 weeks. However, only seven mice were counted in the tail suspension test, because some fell or climbed on the hook.

2. Experiment 2. Effect of different doses of LPS on depressive-like behaviors in mice

Mice were injected intraperitoneally with saline or LPS. Behavioral tests were performed 24 h after the injection. Mice were divided into four groups: saline group, low-dose LPS (L-LPS, 10 µg/kg) group, medium-dose LPS (M-LPS, 1 mg/kg) group, and high-dose LPS (H-LPS, 5 mg/kg) group. Each group had eight mice, aged 8–10 weeks.

3. Experiment 3. Effect of low-dose LPS preconditioning on depressive-like behaviors and microglial activation in mice

Mice received a pretreatment with saline or low-dose LPS (10 μg/kg) on day 0, and were re-challenged with saline or LPS (1 mg/kg) on day 3. Behavioral tests were performed 24 h after the second injection. Mice were divided into four groups: SS group (saline–saline), LS group (10 µg/kg LPS–saline), SL group (saline–1 mg/kg LPS) and LL group (10 µg/kg LPS–1 mg/kg LPS). Each group had eight mice, aged 8–10 weeks.

#### 2.4.2. In Vitro Experiments

1. Experiment 4. Effect of different doses of LPS treatment on microglial activation in vitro

BV2 microglia were seeded in 6-well plates with a density of 5 × 10^5^ cells/well and rested for 24 h. Then, cells were treated with 10 ng/mL, 100 ng/mL, and 1 μg/mL LPS. RNA and protein were collected at indicated times.

2. Experiment 5. Effect of low-dose LPS pretreatment on microglial activation in vitro

BV2 microglia were seeded in 6-well plates with a density of 5 × 10^5^ cells/well and rested for 24 h. Then, cells were pretreated with low-dose LPS (10 ng/mL) for 24 h, followed by washout of LPS with PBS. Cells were rested for an additional 24 h prior to being restimulated with LPS (100 ng/mL)/ Zym (10 μg/mL)/ β-glu (100 μg/mL). RNA and protein were collected at indicated times, after the final stimulation.

The cells were divided into four groups: the PP group (PBS–PBS), LP group (10 ng/mL LPS–PBS), PL group (PBS–100 ng/mL LPS), and LL group (10 ng/mL LPS–100 ng/mL LPS). The rest of the experiments were grouped in a similar manner.

3. Experiment 6. Effect of 6-OAU on low-dose LPS-pretreated microglia in vitro

BV2 microglia were seeded in 6-well plates with a density of 1 × 10^5^ cells/well and rested for 24 h. Then, cells were pretreated with low-dose LPS (10 ng/mL) for 24 h, followed by washout of LPS with PBS. Next, cells were treated with 6-OAU (10 μM or 50 μM) for an additional 24 h. After this, the cells were restimulated with LPS (100 ng/mL) accompanied by 6-OAU. RNA and protein were collected at indicated times, after the final stimulation.

### 2.5. Behavioral Tests 

#### 2.5.1. Open-Field Test (OFT)

The open-field arena (50 × 50 × 30 cm) was made up of blue plastic panels. Mice were individually placed in the center of the open-field arena, and allowed to explore for 5 min while recording [47]. The data were analyzed with the EthoVision XT 14 software (Noldus Co., Wageningen, The Netherlands).

#### 2.5.2. Tail-Suspension Test (TST)

Mice were suspended in the middle of the three-walled rectangular compartment. A metal suspension bar, used to suspend the tail of each mouse, was positioned about 50 cm above the ground. The mice were stuck with a piece of medical tape at the very end of the tail, with about 1 cm of the tail remaining outside the tape. The camera was allowed to record the behavioral changes continuously for 6 min, and the last 4 min of the video were used to analyze the accumulated time of immobile periods with the EthoVision XT 14 software [48].

#### 2.5.3. Forced-Swimming Test (FST)

Mice were forced to swim individually in a glass beaker (2 L; height: 18.4 cm) filled with water (depth: 12 cm), at a temperature of 25 ± 1 °C. The camera was allowed to record the behavioral changes continuously for 6 min, and the last 4 min of the video were used to analyze the accumulated time of immobile periods with the EthoVision XT 14 software [48]. The immobility time was defined as immobile time spent by the mice floating in the water with no active movements, but movements that were necessary to keep their heads above the water.

### 2.6. Cell Viability Assay

BV2 microglia were seeded in 96-well plates with a density of 1 × 10^4^ cells/well. Cells were administrated with the indicated drugs for 24 h, followed by 10 μL cell-counting kit-8 solution (CCK-8; C0037, Beyotime, Shanghai, China) adding to the culture medium, following the manufacturer’s recommendations. The plate was further incubated for 2 h, and the optical density (OD) of the wells was determined using a microplate reader (PowerWave XRS, BioTek, Winooski, VT, USA) at a wavelength of 450 nm. The cell viability was calculated according to the instructions.

### 2.7. ELISA

Cell-free supernatants were collected from BV2 microglia cultures and analyzed for TNF-α (SEKM-0034, Solarbio, Beijing, China) and IL-6 (SEKM-0007, Solarbio) with ELISA kits, according to the manufacturer’s instructions.

### 2.8. Flow Cytometry

Cells were treated according to experimental requirements. After treatment, the cells were washed once with DPBS and the supernatant was carefully aspirated with a vacuum pump. Then pre-warmed 0.25% trypsin was applied to digest cells at 37 °C in an incubator for 1 min. The BV2 microglia medium was added with gentle pipetting, to terminate the digestion, and centrifuged at 1200 rpm at room temperature for 3 min. The supernatant was discarded and the cell pellet resuspended with a rat Fc block (CD16/CD32, 1:100, 101320, Biolegend, San Diego, CA, USA) for 10 min on ice. The cells were labeled with I-A/I-E(MHC Ⅱ)-APC (1:80, 107613, Biolegend) and CD163-PE (1:80, 155307, Biolegend) for 15 min at 4 °C, protected from light. The antibodies were washed out and the cells resuspended with FACS buffer for cytometry analysis with BD LSRFortessa (BD Biosciences). The data were analyzed by designing gates with the Flowjo X 10.0.7 R2 software.

### 2.9. Western Blot

The cells and the brain tissue were lysed by the RIPA buffer (89901, Thermo, CA, USA) containing 1% protease inhibitor cocktail (78439, Thermo, CA, USA) at 4 °C. The supernatant was collected and the concentration of the protein was measured by a BCA protein assay kit (23250, Thermos, CA, USA), according to the manufacturer’s instructions. Protein extracts were separated by 10% SDS-PAGE, and transferred to 0.2 µm nitrocellulose membranes (66485, Pall, New York, NY, USA). Then, the membranes were blocked with 5% milk for 1 h at room temperature, and incubated with primary antibodies (Supplementary Table S1) at 4 °C overnight. After incubation with HRP-conjugated secondary antibodies (Supplementary Table S2) for 1 h at room temperature, the protein bands were detected using enhanced chemiluminescence reagents (32106, Pierce Biotechnology, Rockford, IL, USA). The optical density was measured with ImageJ.

### 2.10. Immunohistochemistry Staining

Mice were anaesthetized with 1.25% tribromoethanol and perfused with cold physiological saline and 4% paraformaldehyde (PFA). After dehydration in 30% sucrose, brains were sectioned in the coronal plane using a freezing microtome. The 30 μm thick sections were obtained for further staining. For immunohistochemistry, brain sections were incubated with 3% H_2_O_2_ to eliminate endogenous peroxidase activity. In order to expose antigen sites for antibodies to bind, quick antigen retrieval solution (C1035, Solarbio, Beijing, China) was applied at room temperature for 5 min. Then, sections were permeabilized for 1 h in PBS with 0.2% Triton-X 100 and 10% BSA at room temperature. After permeabilization, the frozen sections were incubated with goat anti-Iba1 antibody (1:100, ab5076, Abcam, Cambridge, UK) at 4 °C overnight. On the following day, the sections were washed three times with PBS. Following three-time washes with PBS, the sections were incubated with biotinylated secondary antibody mouse anti-goat IgG (sc-2489, Santa Cruz, Dallas, TX, USA) at room temperature for 1 h. To detect the secondary biotinylated antibodies, an amplification system, based on the streptavidin–biotin complex (SABC-HRP Kit; P0603, Beyotime, Shanghai, China), was used afterwards for 30 min. The sections were washed again with PBS, and immunostaining was visualized using 3,3’ diaminobenzidine tetrahydrochloride (DAB; ZLI-9017, ZSGB-Bio, Beijing, China). The sections were rinsed with PBS and the samples mounted permanently with PVP-mounting media. Images were captured using a Leica Aperio VESA8 microscope, and then processed by Aperio Imagescope (Nussloch, Germany). Quantitative analyses were done using ImageJ.

### 2.11. RNA Isolation and Real-Time PCR Quantification

Total RNA from BV2 cells and brain tissues was extracted using the TRIzol reagent (DP424, Tiangen, Beijing, China), according to the manufacturer’s instructions. The synthesis of cDNA was generated from 1ug of total RNA with the Hifair^®^ 1st Strand cDNA Synthesis SuperMix (11123ES60, Yeasen, Shanghai, China). The level of target mRNA in the samples was quantified using a real-time PCR (RT-PCR) based on Hieff^®^ qPCR SYBR^®^ Green Master Mix (Low Rox Plus) (11202ES60, Yeasen, Shanghai, China), according to the manufacturer’s instructions. The PCR reaction was carried out in a QuantStudio 6 Flex instrument (Applied Biosystems, Waltham, MA, USA). The expression of the target genes was normalized to the expression of GAPDH and calculated using the 2^−ΔΔCt^ method. The primer sequences used are summarized in Supplementary Table S3.

### 2.12. RNA-Seq Analysis

Total bulk RNA was extracted from mouse hippocampus using the TRIzol reagent (DP424, Tiangen), followed by library construction using DNA Nanoball (DNB)-making method by BGI. Libraries were sequenced according to paired-end 150 bp base reads, using the DNBSEQ platform. Quality control was performed using SOAPnuke (v1.5.2) to generate fastq files. Reads were aligned to the GPCm38 human reference genome. Read-mapping was performed using Bowtie2 (v2.2.5), and RPKM (reads per kilobase of transcript per Million mapped reads) values of genes were generated by RSEM (v1.2.8) to evaluate the expression level of genes. Differential expression analysis was performed using DESeq2 with adjusted *p*-value ≤ 0.05 and Log2 fold-change > 2. Principal component analysis (PCA) was performed using pheatmap and gene ontology (GO)/ KEGG (Kyoto Encyclopedia of Genes and Genomes) pathway-enrichment analysis was performed using phyper. Other analyses were performed on the website (https://biosys.bgi.com, accessed on 1 September 2022) of BGI.

### 2.13. Statistical Analysis

All data were shown as the mean ± SEM. Comparisons between different groups were made using a one-way ANOVA, followed by a Tukey’s multiple comparisons test with a single-pooled variance. ns: non-significant; * *p* < 0.05; ** *p* < 0.01; *** *p* < 0.001; **** *p* < 0.0001. Statistical analysis was carried out using the GraphPad Prism 9 software (Inc. La Jolla, CA, USA).

## 3. Results

### 3.1. Experimental Results

#### 3.1.1. Low-Dose LPS Did Not Cause a Significant Inflammatory Response in the Hippocampus or Depressive-like Behaviors

Repeated LPS challenges induced immune tolerance phenotype in microglia [49]. To confirm the effect of repeated LPS challenges in inhibiting inflammation, we treated mice with high doses of LPS (5 mg/kg, twice, 6 days apart). Behavioral tests were carried out 3 h after the second LPS injection (Supplementary Figure S1A). In the OFT, the total distance traveled and velocity were reduced in the LPS group compared to the Ctrl group, but were significantly higher in the RL group than in the LPS group (Supplementary Figure S1B–D). In the TST and FST, the immobility time of the LPS-treated mice was increased compared to that of the Ctrl-group mice, while the immobility time of the RL-group mice was significantly decreased compared to that of the LPS-group mice (Supplementary Figure S1E,F). 

Then, the expression levels of *TNF-α*, *IL-1β*, and *IL-6* mRNA in the mouse hippocampus were measured by RT-qPCR. The results showed that a single 5 mg/kg dose of LPS significantly increased the expression of the three proinflammatory cytokines, but that the expression levels of these cytokines were decreased after repeated LPS challenges compared to after a single treatment (Supplementary Figure S1G–I). These results suggest that repeated LPS treatment can suppress inflammation in the brain.

**Figure 1:**
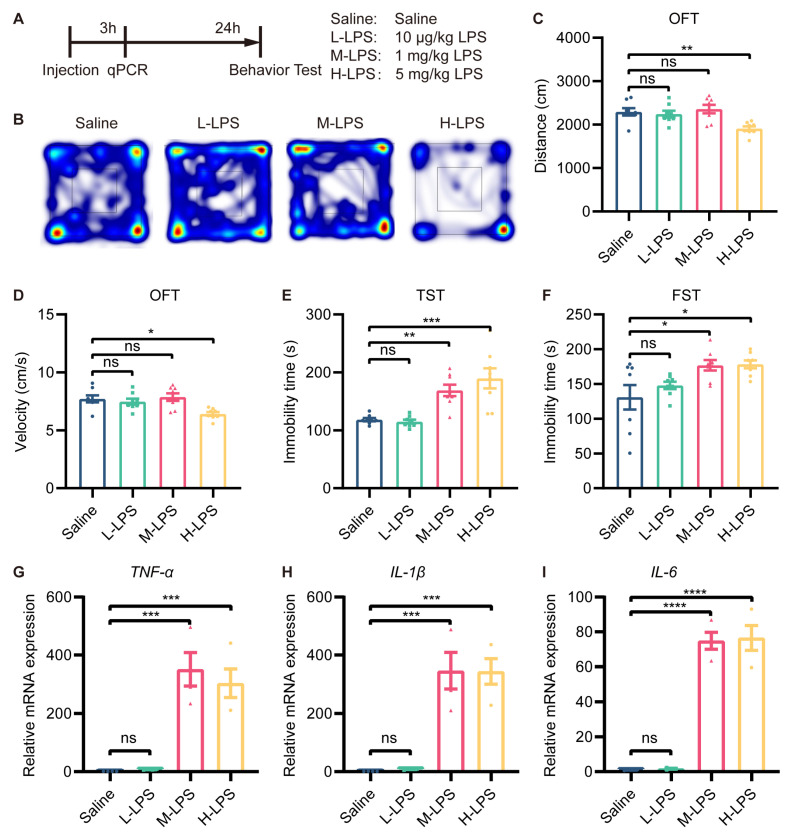
Low-dose LPS did not induce depressive-like behavior in mice. (**A**) Schema of the experiments. Different doses of LPS were administered to induce depressive-like behavior in mice. Mice were injected intraperitoneally with 10 μg/kg, 1 mg/kg or 5 mg/kg LPS, and behavioral tests were performed 24 h after injection (n = 8). Depressive-like behaviors were evaluated by measuring the total distance traveled (**B**,**C**) and velocity (**D**) in the OFT, and immobility time in the TST (**E**) and FST (**F**). (**G**–**I**) mRNA expression levels of *TNF-α*, *IL-1β*, and *IL-6* in the mouse hippocampus were assessed (n = 4). The data were presented as the mean ± SEM; one-way ANOVA, ns indicated no significance, * *p* < 0.05; ** *p* < 0.01; *** *p* < 0.001; **** *p* < 0.0001.

Therefore, we aimed to identify a subthreshold dose of LPS that did not induce depressive-like behavior for preconditioning. To this end, we first treated mice with different doses of LPS (10 μg/kg, 1 mg/kg, and 5 mg/kg), observed their behavior and measured the level of inflammation (Figure 1A). In the OFT, there was no difference in the total distance traveled or velocity among the saline-, low-dose LPS (L-LPS, 10 µg/kg)- and medium-dose LPS (M-LPS, 1 mg/kg)-treated mice. However, the total distance traveled and velocity were reduced in the high-dose LPS (H-LPS, 5 mg/kg)-treated mice compared to the saline-treated mice (Figure 1B–D). In the TST and FST, L-LPS-treated mice showed no difference in immobility time compared to saline-treated mice, while M-LPS- and H-LPS-treated mice showed a significant increase in immobility time (Figure 1E,F). 

RT-qPCR revealed that L-LPS treatment did not alter the mRNA expression of *TNF-α*, *IL-1β*, and *IL-6* in the hippocampus, whereas M-LPS treatment did (Figure 1G–I). In addition, compared with M-LPS, H-LPS did not further increase the mRNA expression of *TNF-α*, *IL-1β* or *IL-6* in the hippocampus (Figure 1G–I). Therefore, we can conclude that L-LPS does not cause a significant inflammatory response in the hippocampus or depressive-like behavior in mice.

#### 3.1.2. Low-Dose LPS Preconditioning Exerted Antidepressant-like Effects in Inflammation-Induced Depressive-like Behavior

**Figure 2:**
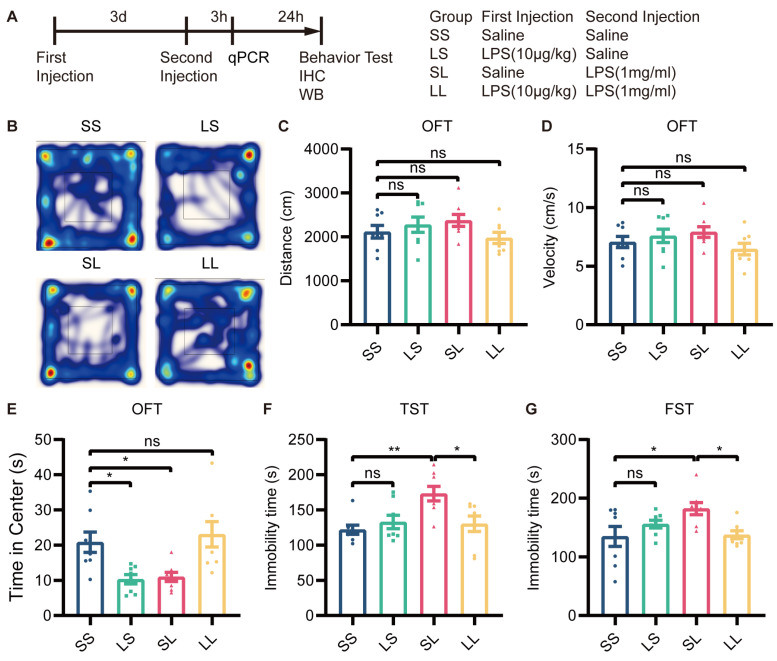
Low-dose LPS preconditioning exerted antidepressant-like effects. (**A**) Experimental schema. Low-dose LPS (10 μg/kg) or saline was administered before a second injection of high-dose LPS (1 mg/kg) or saline 3 days later, and behavioral tests were performed 24 h after the second injection (n = 8). Depressive-like behaviors were evaluated by measuring the total distance traveled (**B**,**C**), velocity (**D**), and total time spent at the center (**E**) in the OFT, and immobility time in the TST (**F**) and FST (**G**). The data were presented as the mean ± SEM; one-way ANOVA, ns indicated no significance, * *p* < 0.05; ** *p* < 0.01.

To determine whether L-LPS preconditioning blocks depressive-like behavior induced by M-LPS, we pretreated mice with 10 µg/kg LPS and treated them 3 days later with 1 mg/kg LPS. Behavioral tests were conducted 24 h after the last stimulation (Figure 2A). In the OFT, there was no significant difference in the total distance traveled or velocity among the four groups (Figure 2B–D). Mice in the LS and SL groups spent less time in the central area than mice in the SS group; however, mice in the LL group spent more time in the central area than mice in the SL group (Figure 2B,E). In the TST and FST, the immobility time of the LS-group mice did not differ from that of SS-group mice, while SL-group mice showed a significant increase in immobility time; however, immobility time was reduced in LL-group mice compared to SL-group mice (Figure 2F,G). According to these findings, L-LPS preconditioning exerted antidepressant-like effects in inflammation-induced depression.

#### 3.1.3. Low-Dose LPS Preconditioning Repressed Microglial Activation in an Inflammation-Induced Depression Mouse Model

**Figure 3:**
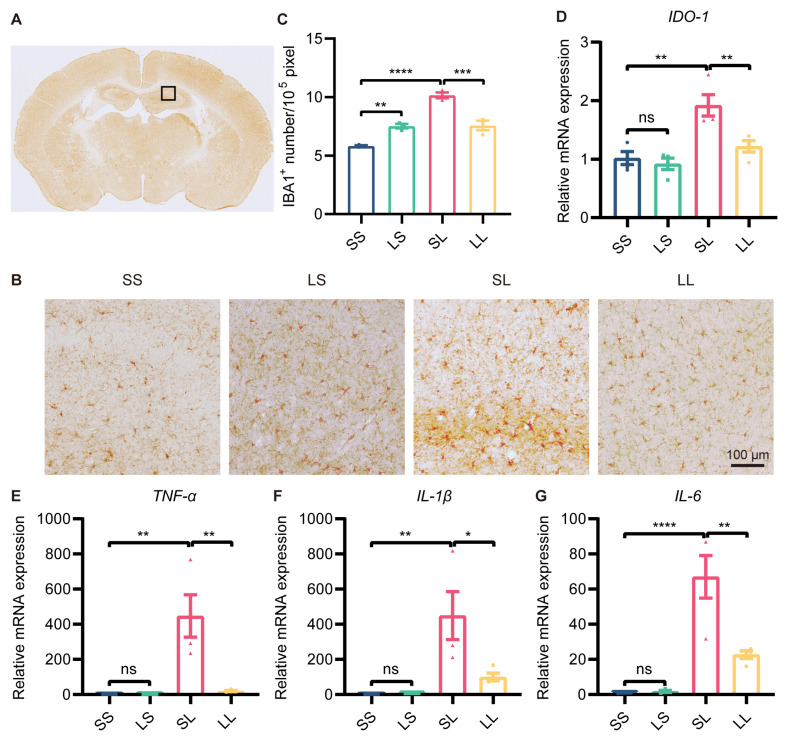
Effect of low-dose LPS preconditioning on microglial activation. (**A**) Schematic representation of the target brain region. (**B**) Immunohistochemical staining of Iba1 in the hippocampus. Representative images were shown; scale bar = 100 μm. (**C**) Quantitative analysis of Iba1-positive cell numbers in the hippocampus (n = 3). (**D**–**G**) mRNA expression levels of *IDO1, TNF-α*, *IL-1β*, and *IL-6* in the mouse hippocampus (n = 4). The data were presented as the mean ± SEM; one-way ANOVA, ns indicated no significance, * *p* < 0.05; ** *p* < 0.01; *** *p* < 0.001; **** *p* < 0.0001.

A growing body of research suggested that depression was often accompanied by microglial overactivation. We then detected changes in microglial activation in the hippocampus by immunohistochemical staining for Iba1 (Figure 3A). The results showed that the number of Iba1^+^ cells was increased in the LS group compared to the SS group, and was further increased in the SL group; however, the number of Iba1^+^ cells was decreased in the LL group compared to the SL group (Figure 3B,C). It was well known that *IDO1* expression was a characteristic of MDD pathology. To evaluate the effect of L-LPS preconditioning on *IDO1* expression, we examined *IDO1* expression in the hippocampus by RT-qPCR. The mRNA expression of *IDO1* was not changed in the LS group compared to the SS group, but was increased in the SL group compared to the SS group; however, *IDO1* expression was decreased in the LL group compared to the SL group (Figure 3D). In addition, the expression of BDNF and synapse-associated proteins in the hippocampus was measured. Western blotting showed that the protein expression of BDNF, synaptophysin, and PSD95 in the mouse hippocampus was not different among the four groups (Supplementary Figure S2A–D). Furthermore, the mRNA expression of *TNF-α*, *IL-1β*, and *IL-6* was increased in the SL-group mice compared to the SS-group mice, but decreased in the LL-group mice compared to the SL-group mice (Figure 3E–G). According to these findings, low-dose LPS preconditioning exerted an anti-inflammatory effect in inflammation-induced depression.

#### 3.1.4. Low-Dose LPS Pretreatment Decreased the Secondary Stimulation-Induced Inflammatory Response in BV2 Microglia

**Figure 4:**
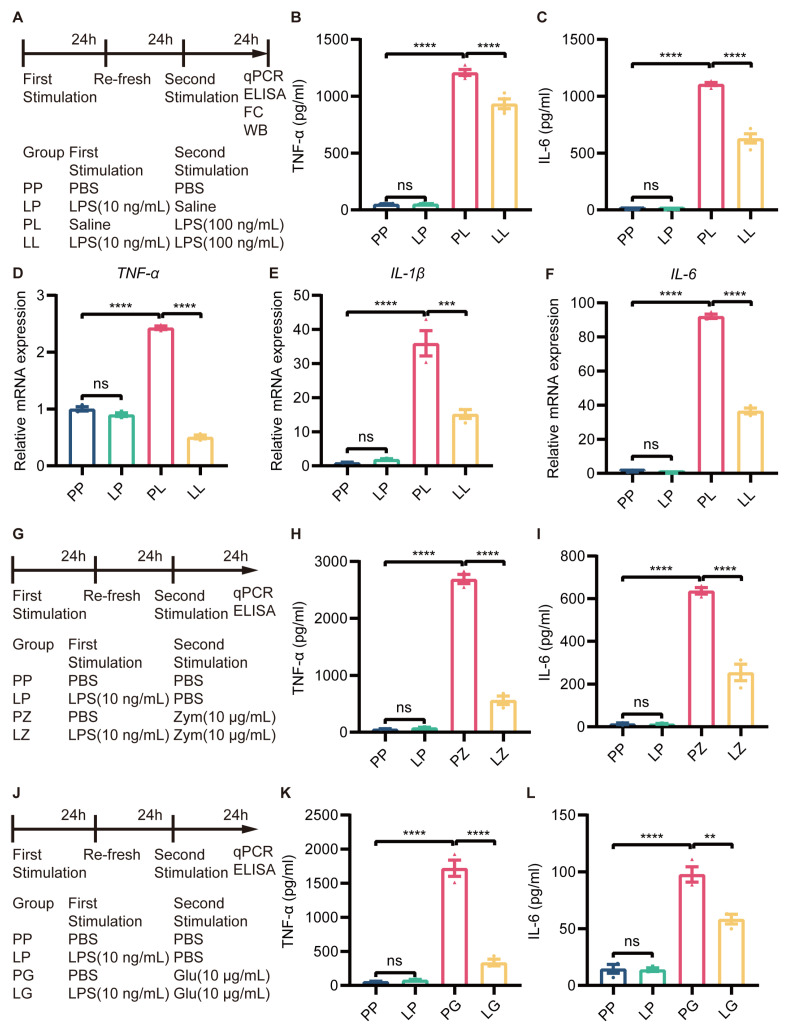
Low-dose LPS pretreatment decreased the secondary stimulation-induced inflammatory response in BV2 microglia. (**A**) Experimental schema. Low-dose LPS (10 ng/mL) or PBS was applied before administration of a second high dose of LPS (100 ng/mL) or PBS. The cell culture supernatant was collected to measure TNF-α (**B**) and IL-6 (**C**) protein expression using ELISA kits (n=4). mRNA expression levels of *TNF-α* (**D**), *IL-1β* (**E**), and *IL-6* (**F**) were quantified 24 h after the second LPS stimulation in BV2 microglia (n = 3). (**G**,**J**) Experimental schema. Cells were subjected to secondary stimulation with Zym or β-glu rather than LPS. The protein expression of TNF-α (**H**,**K**) and IL-6 (**I**,**L**) was measured via ELISA 24 h after the second stimulation. The data were presented as the mean ± SEM; one-way ANOVA, ns indicated no significance,** *p* < 0.01; *** *p* < 0.001; **** *p* < 0.0001.

The inflammatory response induced by LPS in BV2 microglia was dose-dependent, with only 1 µg/mL LPS being cytotoxic (Supplementary Figure S3). To establish endotoxin tolerance in BV2 microglia, the cells were treated with 10 ng/mL LPS and then subsequently stimulated with 100 ng/mL LPS (Figure 4A). Then, the immunosuppressive effect of low-dose LPS pretreatment was assessed by measuring the expression of proinflammatory cytokines via ELISA and RT-qPCR. The cell culture supernatant was collected to test secretory protein expression by ELISA, and the results showed that the expression of TNF-α and IL-6 was not changed in LP-treated BV2 microglia, but increased in PL-treated BV2 microglia compared to PP-treated microglia. Surprisingly, decreased expression of TNF-α and IL-6 was observed in the LL group compared to the PL group (Figure 4B,C). RT-qPCR revealed that the changes in *TNF-α*, *IL-1β*, and *IL-6* expression were consistent with the changes in protein expression described above (Figure 4D–F). In addition, to determine whether tolerance-like microglia were only hyporesponsive to LPS, we restimulated LPS-pretreated microglia with zymosan and β-glucose (Figure 4G,J). The changes in TNF-α and IL-6 protein expression were consistent with those observed in the LL group (Figure 4H,I,K,L). The mRNA expression of *TNF-α*, *IL-1β*, and *IL-6* was also decreased in tolerance-like BV2 microglia after treatment with zymosan (Supplementary Figure S4) and β-glucose (Supplementary Figure S5). These results suggested that low-dose LPS pretreatment transforms microglia to a hyporeactive state, in which they were not tolerant to LPS.

#### 3.1.5. Low-Dose LPS Pretreatment Decreased M1 Polarization and ERK Phosphorylation in BV2 Microglia under Inflammatory Conditions

**Figure 5:**
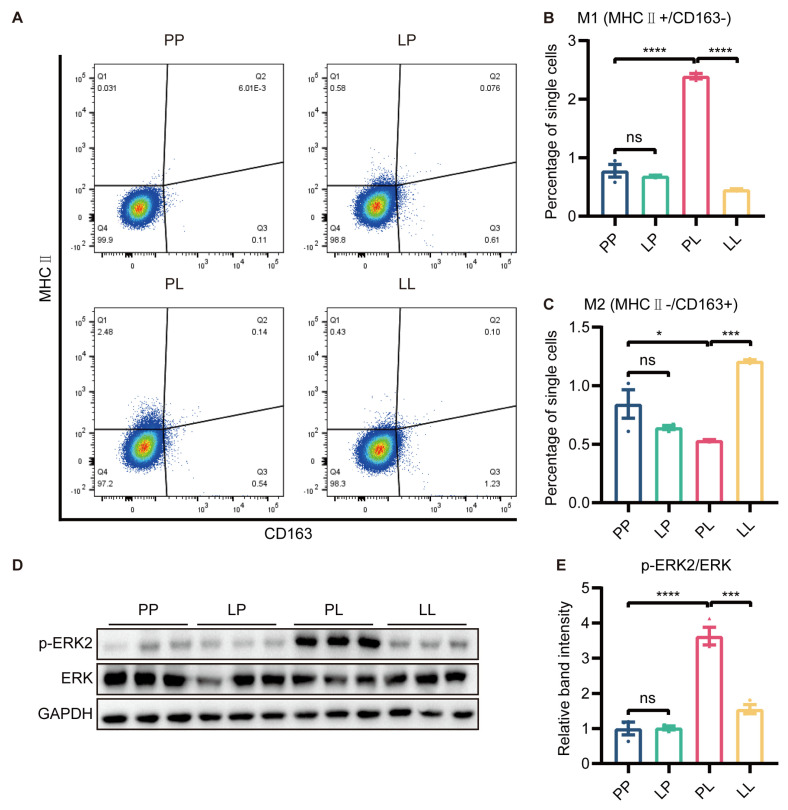
The effect of low−dose LPS pretreatment on phenotypic transition in BV2 microglia. (**A**) Representative graph of flow cytometry results. MHC Ⅱ and CD163 were used to identify M1 and M2 microglia, respectively. MHC Ⅱ (**B**) and CD163 (**C**) protein expression was measured via flow cytometry. M1 microglial polarization was reduced and M2 microglial polarization was promoted in the LL group compared to the PL group (n = 3). (**D**) Representative Western blots of p−ERK2 and ERK in BV2 microglia; the level of each protein was normalized to the GAPDH level (n = 3). (**E**) Statistical analysis showed that the phosphorylation of the ERK protein in BV2 microglia was decreased in the LL group compared to the PL group. The data were presented as the mean ± SEM; one-way ANOVA, ns indicated no significance, * *p* < 0.05; *** *p* < 0.001; **** *p* < 0.0001.

It was well known that M1 polarization of microglia promoted the development of inflammatory responses. To evaluate whether low-dose LPS pretreatment affected microglial polarization, we measured the expression of MHC Ⅱ and CD163 through flow cytometry in BV2 cells. The polarization of cells was not changed in the LP group, and microglial polarization to the M1 (MHC Ⅱ^+^, CD163^−^) phenotype was promoted in the PL group. However, the protein expression of MHC Ⅱ was markedly downregulated and CD163 expression was greatly increased by low-dose LPS pretreatment in the LL group compared to the PL group (Figure 5A–C and Supplementary Figure S6).

To further investigate why low-dose LPS pretreatment switched microglia from the M1 to M2 phenotype, we examined the protein expression of ERK and p-ERK using Western blotting. The phosphorylation of ERK protein in BV2 microglia was higher in the PL group than in the PP group, while decreased p-ERK immunoreactivity was detected in BV2 microglia in the LL group (Figure 5D,E). ERK signaling was involved in low-dose LPS-triggered microglial phenotype switching.

#### 3.1.6. Changes in the Transcriptional Profile of Tolerance-like Mice

**Figure 6:**
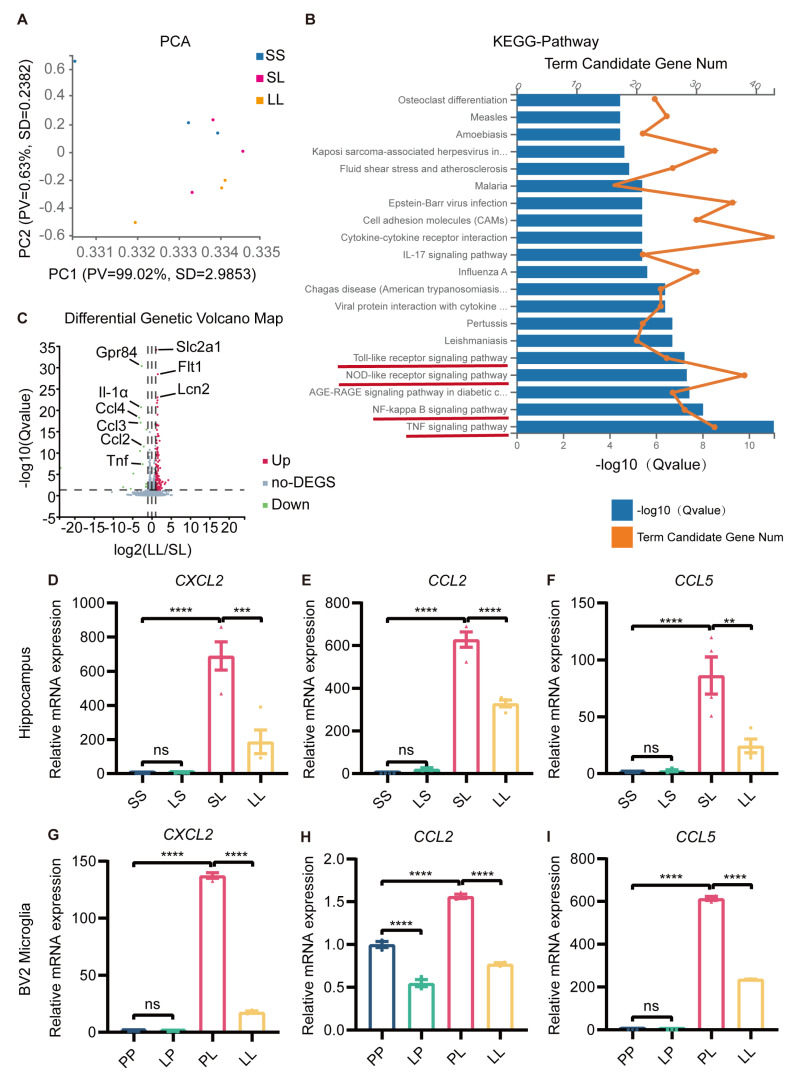
Transcriptional profile changes in tolerance-like mice. (**A**) PCA separated the sample into three clusters (SS, SL, LL), which coincided with the real situation (n = 3). (**B**) KEGG pathway analysis of differentially expressed genes between the SL and LL groups. (**C**) Volcano plot of differentially expressed genes between the SL and LL groups. Genes above the significance threshold (Padj < 0.05, Log2 FC > 2) were colored green and red. (**D**–**F**) mRNA expression levels of *CXCL2*, *CCL2*, and *CCL5* in the mouse hippocampus (n = 4). (**G**–**I**) mRNA expression levels of *CXCL2*, *CCL2*, and *CCL5* in BV2 microglia (n=3). The data were presented as the mean ± SEM; one-way ANOVA, ns indicated no significance, ** *p* < 0.01; *** *p* < 0.001; **** *p* < 0.0001.

So far, we had revealed the antidepressant and anti-inflammatory effects of low-dose LPS pretreatment, but the mechanism was still unclear. Hippocampal RNA from mice in the SS, SL, and LL groups was used for transcriptome sequencing. PCA clustering analysis showed that the transcriptome profiles of the three groups were distinct from each other (Figure 6A). Next, we performed KEGG pathway analysis of the differentially expressed genes (DEGs) between SL and LL groups, which revealed that a large number of genes were enriched in the toll-like receptor signaling pathway, nod-like receptor signaling pathway, NF-κB signaling pathway, and TNF signaling pathway (Figure 6B). GO-C-enrichment analysis revealed that altered genes were enriched in the cytoplasm, plasma, and membrane (Supplementary Figure S7A). Volcano plots revealed differences in gene expression between the SL and LL groups, and indicated that the expression of *GPR84* and chemokines was downregulated in the LL group compared to the SL group (Figure 6C). Analysis of the heatmap showed that the SL and LL groups had different expression patterns (Supplementary Figure S7B). To further validate the sequencing results, the expression of chemokines in the hippocampus of low-dose LPS-pretreated mice and BV2 microglia was examined. The results showed that low-dose LPS pretreatment reduced the expression of the chemokines *CXCL2*, *CCL2* and *CCL5*, which was induced by LPS (Figure 6D–I). The RNA sequencing results further demonstrated that low-dose LPS preconditioning reduced the inflammatory response in the brain upon re-exposure to detrimental stimuli, which was beneficial in preventing the development of excessive inflammation and protecting the organism.

#### 3.1.7. GPR84 Mediated LPS-Induced Microglial Tolerance-like State

**Figure 7:**
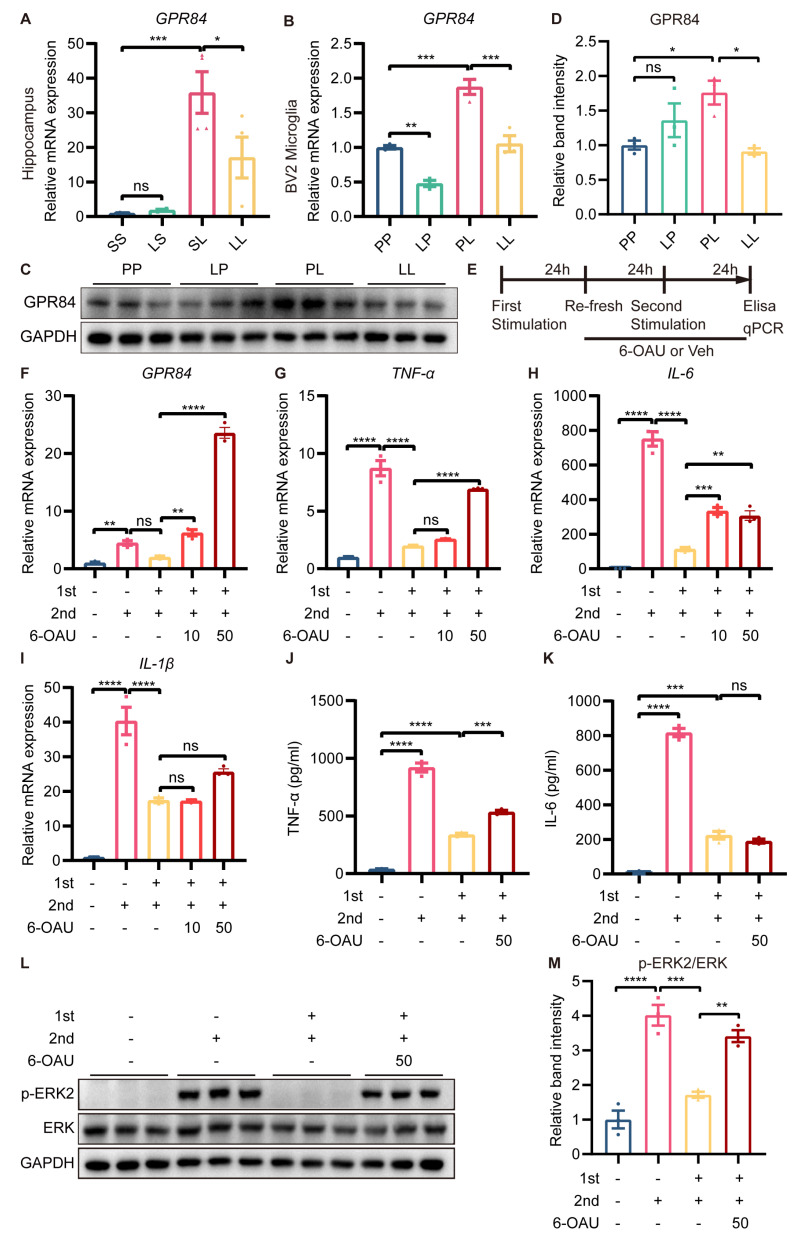
Effect of GPR84 activation on LPS−induced microglial tolerance−like state. *GPR84* mRNA expression in the mouse hippocampus (n = 4) (**A**) and in BV2 microglia (n = 3) (**B**). (**C**) Representative Western blots of GPR84 and the normalization control GAPDH in BV2 microglia. (**D**) Statistical analysis showed that the expression of GPR84 protein in BV2 microglia was increased in the PL group compared to the PP group, but decreased in the LL group compared to the PL group (n = 3). (**E**) Schematic diagram of the 6−OAU treatment experiment. “+” referred to LPS treatment, “-“ referred to saline/vehicle treatment, “10” referred to 10 μM, and “50” referred to 50 μM. (**F**–**I**) mRNA expression levels of *GPR84*, *TNFα*, *IL−6* and *IL−1β* in BV2 microglia. (**J**,**K**) Protein levels of TNFα and IL-6 in the cell culture supernatant (n = 3). (**L**) Representative Western blots of p−ERK2 and ERK in BV2 microglia; the level of each protein was normalized to the GAPDH level (n = 3). (**M**) Statistical analysis showed that the phosphorylation of the ERK protein in BV2 microglia was increased in the LL + 6OAU group compared to the LL + vehicle group. The data were presented as the mean ± SEM; one−way ANOVA, ns indicated no significance, * *p* < 0.05; ** *p* < 0.01; *** *p* < 0.001; **** *p* < 0.0001.

We were intrigued by the fact that GPR84, which was essential for inflammation, was expressed at lower levels in the LL group than in the SL group. Therefore, further experiments were carried out to confirm the changes in GPR84 expression. RT-qPCR showed that the expression of *GPR84* was significantly lower in the hippocampus of mice in the LL group than in the hippocampus of mice in the SL group (Figure 7A). In vitro, the mRNA expression of *GPR84* in BV2 microglia was decreased in the LP group compared to the PP group (Figure 7B), although the protein levels of GPR84 were not changed in the LP group (Figure 7C,D). The mRNA and protein expression of GPR84 in BV2 microglia was increased in the PL group compared to the PP group (Figure 7B–D); however, GPR84 expression in BV2 microglia was decreased in the LL group compared to the PL group (Figure 7B–D).

Having found that GPR84 expression was downregulated in tolerance-like microglia, we wanted to explore whether GPR84 was essential for LPS-induced microglial tolerance-like state. A selective agonist of GPR84, namely 6-n-octylaminouracil (6-OAU), was used to block LPS-induced microglial tolerance-like state (Figure 7E). RT-qPCR showed that 10 μM and 50 μM 6-OAU treatment increased the expression levels of *GPR84* mRNA compared to tolerance-like microglia (Figure 7F). In addition, 10 μM 6-OAU restored *IL-6* mRNA expression levels, but had no effect on *TNF-α* and *IL-1β* mRNA expression; 50 μM 6-OAU increased *TNF-α* and *IL-6* mRNA expression, but still had no effect on *IL-1β* mRNA expression (Figure 7G–I). To confirm this, BV2 microglia were treated with 50 μM 6-OAU, and the protein expression of *TNF-α* and *IL-6* in the cell culture medium was examined. The results showed that 6-OAU increased TNF-α protein expression, but had no effect on IL-6 protein expression (Figure 7J,K). Furthermore, 50 μM 6-OAU treatment promoted the phosphorylation of ERK protein in BV2 microglia compared to tolerance-like microglia (Figure 7L,M). Thus, we can conclude that GPR84 reactivation partially abrogated LPS-induced microglial tolerance-like state, suggesting that GPR84 played a role in modulating the microglial phenotype shift, but may not be the sole cause of microglial tolerance-like state.

Microglial overactivation, and subsequently severe neuroinflammation, promoted the development of depression. Low-dose LPS preconditioning, which restricted microglial overactivation and alleviated inflammation-induced depressive-like behavior in mice, can be used as a powerful strategy for depression treatment.

## 4. Discussion

In the present study, a low dose of LPS (10 µg/kg) was identified to effectively precondition microglia, but was insufficient in inducing depressive-like behavior in mice. Further, the antidepressant effects of low-dose LPS preconditioning were confirmed in a mouse model of inflammation-induced depression. Interestingly, GPR84 was found to be downregulated in the hippocampus of tolerance-like mice in our bulk RNA sequencing study. In vitro, low-dose LPS preconditioning induced a microglial tolerance-like state, which could be partially abrogated by a GPR84 agonist. In summary, low-dose LPS preconditioning alters the immune properties and phenotype of microglia, which can prevent overactivation of microglia and may be neuroprotective against diseases.

MDD is accompanied by severe neuroinflammation, during which cytokines are generated in large amounts, leading to pathological changes, such as neuronal damage [50,51]. LPS preconditioning transforms microglia into a neuroprotective phenotype to maintain homeostasis of the central nervous system [52]. Therefore, LPS preconditioning is a promising strategy to prevent microglial overactivation and severe neuroinflammation. However, intraperitoneal injection of LPS (0.82 mg/kg or 1 mg/kg) induces a strong neuroinflammatory response and depressive-like behavior in mice. Hence, identifying the threshold dose of LPS that is insufficient to induce depressive-like behavior is important. In the present study, mice were intraperitoneally injected with 10 µg/kg, 1 mg/kg, and 5 mg/kg LPS, then their behaviors were observed and inflammation levels measured (Figure 1A). M-LPS (1 mg/kg) and H-LPS (5 mg/kg) induced a significant inflammatory response in the hippocampus and depressive-like behavior in mice (Figure 1). This is consistent with previous studies, in which 0.83 mg/kg LPS and 1 mg/kg LPS were used to induce depression. However, L-LPS (10 µg/kg) did not cause depressive-like behavior (Figure 1). Therefore, in the next experiments, 10 µg/kg LPS was chosen for preconditioning. L-LPS preconditioning indeed decreased neuroinflammation and alleviated depressive-like behavior in a mouse model of inflammation-induced depression (Figure 2). It is clear that this dose of LPS can be used as a microglial modulator to treat neurological disorders. However, further work is needed to confirm whether long-term injection of L-LPS causes damage to the body and whether it has the same effects.

IDO1 is a significant contributor to depression, and inhibiting IDO1 ameliorates depressive-like behaviors in mice [43]. Expression of IDO1 in the hippocampus was increased in SL-treated mice compared to SS-treated mice, which was prevented by L-LPS preconditioning in LL-treated mice (Figure 3D). However, changes in the expression of BDNF and synapse-associated proteins were not observed (Supplementary Figure S2). The above results suggest that low-dose LPS preconditioning may ameliorate inflammation-induced depression by affecting IDO1 expression. The Trp–Kyn metabolic pathway is associated with local immunosuppression in the tumor microenvironment. Moreover, enhanced activity of the Trp–Kyn metabolic pathway leads to tryptophan depletion and accumulation of metabolites, such as kynurenine, promoting immune escape of cancer cells [53]. IDO1 can metabolize tryptophan to kynurenine, thus affecting the Trp–Kyn metabolic pathway. Therefore, the abnormally high expression of IDO1 is closely related to tumor immune escape and is an important target for tumor immunotherapy. The resolution of the mechanism of tolerance-like microglial formation may provide new research perspectives for tumor immunity issues, especially in the brain.

Depression is an extremely complex disease with unclear mechanisms. There is a consensus that microglial activation promotes depression, and that microglial elimination has an antidepressant effect [21,22,25]. However, lower numbers of microglia in the dentate gyrus (DG) are observed in depressed mice. Microglial stimulation plays an antidepressant role in a chronic unpredictable stress (CUS) mouse model [4]. Under stress conditions, microglial tolerance may occur to avoid damage to neurons [54]. Microglial tolerance plays a beneficial role early in depression by limiting microglial activation, but becomes a limiting factor later in the disease when normal microglial function is needed. Therefore, in the CUS mouse model, microglial activation induced by low doses of LPS has an antidepressant effect [4]. PSD occurs in approximately one-third of stroke survivors and is characterized by increased levels of IDO1 in microglia in the brain [44]. Stroke is accompanied by the overactivation of microglia and the release of large amounts of proinflammatory cytokines, which may contribute to PSD. Therefore, we speculate that low-dose LPS preconditioning is a promising strategy for the prevention of PSD.

It is well established that LPS exposure causes innate immune cells to become hyporeactive. However, it is not clear whether microglia in this state are only hyporesponsive to LPS. In the present study, LPS-induced hyporesponsive microglia were rechallenged with LPS, zymosan and β-glucose. The influence of immunosuppression on the effects of these different inflammation inducers was similar. M1/M2 polarization of microglia is related to the ERK signal transduction. In the present study, ERK phosphorylation in microglia was promoted in the PL group and decreased in the LL group (Figure 5D,E). It was further shown that LPS preconditioning exerted anti-inflammatory effects on microglia by affecting ERK signaling. ERK is the key to signal transmission from surface receptors to the nucleus and is directly involved in the activation of a large number of downstream substrates as the only activation site, which in turn induces regulation of cell proliferation, differentiation, and apoptosis by regulating the various proteins transcribed [55]. Abnormal activation of ERK will promote tumor cell growth. All cells, including tumor cells, need to obtain the required nutrients through blood vessels, and without the accompanying vessels, it is difficult for tumors to grow. The abnormal activation of ERK/MAPK pathway can continuously transcribe vascular endothelial growth factor (VEGF), which can accelerate tumor growth by promoting angiogenesis [56,57]. Selective inhibition of ERK reduces inflammation and enhances insulin sensitivity. Therefore, the development of ERK inhibitors is also a critical direction in the field of neurodegenerative disease treatment. Low-dose LPS preconditioning significantly reduces ERK phosphorylation levels induced by subsequent stimulation, and may also have a role in the treatment of tumors and neurodegenerative diseases.

Endotoxin tolerance is an adaptive response of the body that can effectively inhibit excessive inflammation. However, it is not clear how hyperactive microglial cells become hyporeactive after exposure to LPS. In the THP1 human monocyte cell line, myristyl acetate reverses endotoxin tolerance and promotes TNF-α production by activating protein kinase C [58]. The administration of flt3 ligand reverses endotoxin tolerance in dendritic cells, allowing them to produce proinflammatory cytokines once again [59]. GPR84, a member of the metabolic G-protein-coupled receptor family, is expressed primarily in immune cells. GPR84-mediated signaling leads to microglial cell motility and membrane folding, but does not promote the inflammatory response. As GPR84 is a known receptor for medium-chain fatty acids, such fatty acids released from damaged brain cells may be involved in the enhancement of microglial motility through GPR84 after neuronal injury [60]. However, whether GPR84 is involved in the development of endotoxin tolerance is unknown. In this study, GPR84 was found to be downregulated in tolerance-like microglia (Figure 6C and Figure 7A–D). This is the first indication that GPR84 is involved in the development of endotoxin tolerance. Activation of the GPR84 receptor with 6-OAU increases the expression of p-ERK under inflammatory conditions [61,62]. Therefore, in this study, tolerance-like BV2 microglia were treated with 6-OAU prior to secondary stimulation. The results showed that proinflammatory cytokine expression was partially restored and p-ERK expression was increased, indicating that GPR84 activation could reverse the microglial tolerance-like state (Figure 7E–M). Based on the role of GPR84 in modulating the microglial tolerance-like state, further studies may target GPR84 for the treatment of neurological disorders that are highly associated with neuroinflammation, such as Alzheimer’s disease and Parkinson’s disease [63,64].

Inflammatory depression, also known as cytokine-induced depression or immune-related depression, is a subtype of depression. It is believed that increased inflammatory responses, such as inflammatory cytokines, can trigger depressive symptoms in some individuals. This type of depression may be more resistant to traditional antidepressant medications and may require different treatment approaches, such as anti-inflammatory drugs or lifestyle interventions, to reduce inflammation. Tolerance-like microglia are a recently discovered subtype of microglia, which exhibit an altered phenotype, characterized by reduced expression of pro-inflammatory genes and enhanced expression of genes associated with tissue repair and regeneration. In our study, we proved that tolerance-like microglia can limit inflammation and reduce depressive-like behaviors in inflammatory depression. This evidence suggests that low-dose LPS preconditioning may be a better approach to treating inflammatory depression than traditional antidepressants.

Tolerance-like microglia have been observed in various neurological conditions, including multiple sclerosis, Alzheimer’s disease and traumatic brain injuries. However, the current identification of tolerance-like microglia is defined in terms of phenotypic changes in reduced expression of inflammatory factors and lacks its specific antigenic markers. In our research, the GPR84 was found to be significantly downregulated in tolerance-like microglia, and the activation of GPR84 reversed the tolerance-like phenotype of microglia. These results suggest that GPR84 may be a molecular marker for tolerant-like microglia, but further experiments are needed to confirm this.

## 5. Conclusions

In this study, a subthreshold dose of LPS (10 µg/kg) was shown to suppress the inflammatory response and alleviate depressive-like behavior in a mouse model of inflammation-induced depression. This was the first study to find that low-dose LPS preconditioning prevented microglial overactivation and protected mice from depressive-like behavior, possibly by downregulating GPR84 expression. These findings suggest that low-dose LPS preconditioning exerts anti-inflammatory and antidepressant effects in inflammatory depression, by downregulating GPR84 expression, and thereby inhibiting microglial overactivation.

## 6. Limitations and Future Directions

Although this study has addressed the impacts of low-dose LPS preconditioning on microglial activation and inflammatory depression, several limitations also apply. First, the behavior tests were limited. This is due to the limitation of mouse model of inflammation-induced depression, lacking adequate time to do more behavior tests. Secondly, we do not know if low-dose LPS preconditioning is effective in humans. This is because we are not qualified to conduct clinical studies. Future studies will adopt more dimensional evaluation metrics, test the effects on more animal models, and initiate clinical studies at the appropriate time.

## Data Availability

Data reported in this study are available upon reasonable request.

## Supplementary Materials

The following supporting information can be downloaded at: https://www.mdpi.com/article/10.3390/brainsci13040549/s1, Figure S1: Repeated stimulation with 5 mg/kg LPS suppressed the inflammatory response in the brain; Figure S2: The protein expression of BDNF and synapse-associated proteins; Figure S3: The inflammatory response induced by LPS in BV2 microglia was dose-dependent; Figure S4: Low-dose LPS pretreatment induced a tolerance-like response to subsequent Zym challenge in BV2 microglia; Figure S5: Low-dose LPS pretreatment induced a tolerance-like response due to subsequent β-glu challenge in BV2 microglia; Figure S6: Low-dose LPS pretreatment promoted phenotypic transition in BV2 microglia; Figure S7: Transcriptional profile changes in tolerance-like mice; Figure S8: Raw images of the Western blot in Figure 5D, Figure 7D and Figure 7L. Table S1: Primary antibody used in WB; Table S2: Secondary antibody used in WB; Table S3: Primers used in qPCR. Table S4: Raw data for behavioral tests in Figure 1 and Figure 2.
