# Supplementary material for: Lipopolysaccharide Preconditioning Restricts Microglial Overactivation and Alleviates Inflammation-Induced Depressive-like Behavior in Mice
Source: Brain Sci. 2023 Mar 25;13(4):549. doi: 10.3390/brainsci13040549 (PMC10137116; doi:10.3390/brainsci13040549)
Supplement: Supplementary file 1 [file brainsci-13-00549-s001.zip › brainsci-2271554-Supplementary Material.pdf]

Supplementary Material

1 Supplementary Figures and Tables

1.1 Supplementary Figures

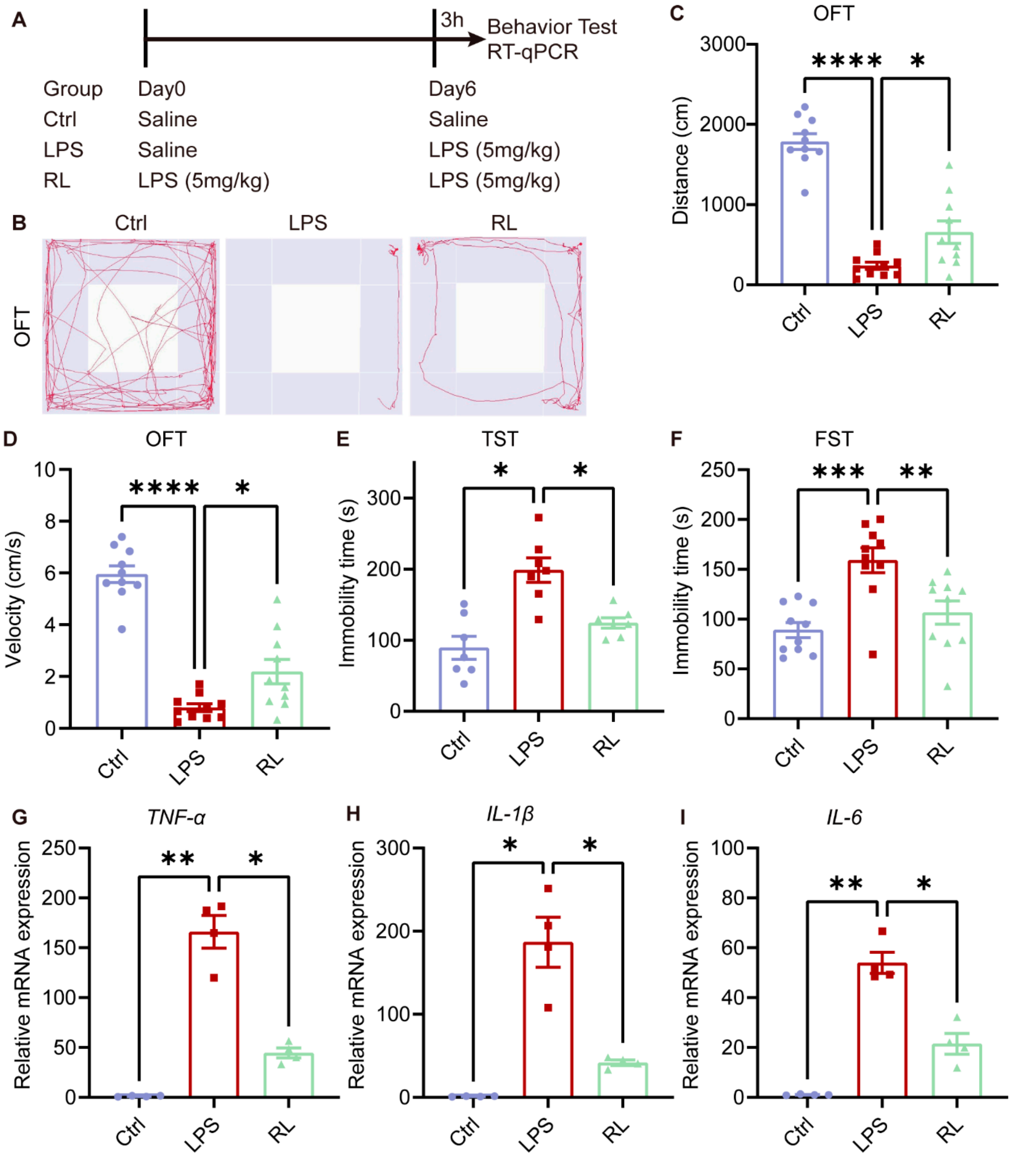

Supplementary Figure S1. Repeated stimulation with 5 mg/kg LPS suppressed the inflammatory response in the brain. **(A)** Schematic of experiments testing the effects of repeated 5 mg/kg LPS treatment on mouse behavior. Mice were repeatedly intraperitoneally injected with 5 mg/kg LPS at an

interval of 6 days, and behavioral tests were performed 3 h after the second injection (n=7-10). Total distance traveled **(B, C)** and velocity **(D)** in the OFT and immobility time in the TST **(E)** and FST **(F)**. **(G-I)** mRNA expression levels of *TNF-α*, *IL-1β*, and *IL-6* in the mouse hippocampus were measured (n=4). The data are presented as the mean ± SEM; one-way ANOVA, ns indicates no significance, *$p < 0.1$, **$p < 0.01$, ***$p < 0.001$, ****$p < 0.0001$.

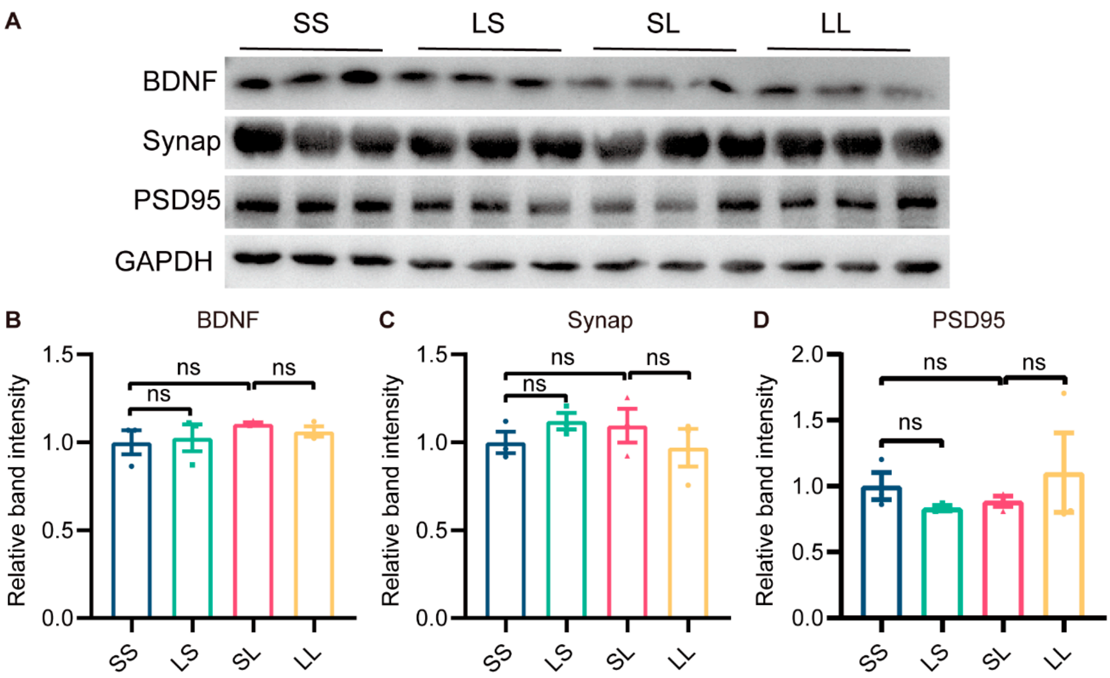

Supplementary Figure S2. The protein expression of BDNF and synapse-associated proteins. **(A)** Representative western blots of BDNF, Synap, PSD95 and the normalization control GAPDH in the hippocampus (n=3). **(B-D)** Statistical analysis showed that neither low-dose nor high-dose LPS had an effect on hippocampal BDNF, synap, and PSD95 protein expression. The data are presented as the mean ± SEM; one-way ANOVA, ns indicates no significance.

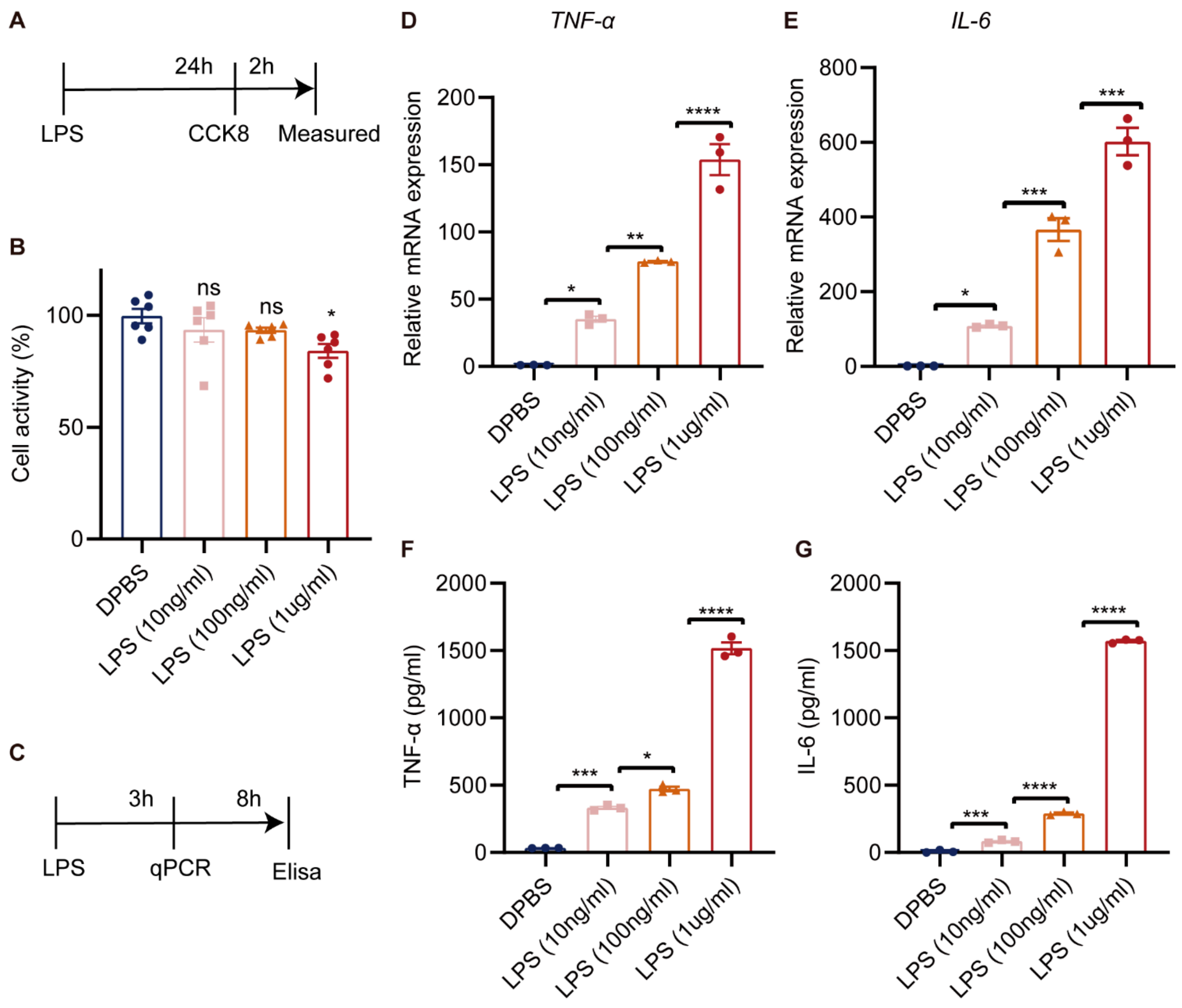

Supplementary Figure S3. The inflammatory response induced by LPS in BV2 microglia was dose-dependent. **(A)** Schematic diagram of the CCK8 assay. **(B)** Cell viability was quantified using the CCK8 assay (n=6). **(C)** Schematic diagram of the LPS stimulation experiment. **(D, E)** mRNA expression levels of *TNFα* and *IL-6* in BV2 microglia (n=3). **(F, G)** Protein levels of TNFα and IL-6 in the cell culture supernatant (n=3). The data are presented as the mean ± SEM; one-way ANOVA, ns indicates no significance, *$p < 0.1$, **$p < 0.01$, ***$p < 0.001$, ****$p < 0.0001$.

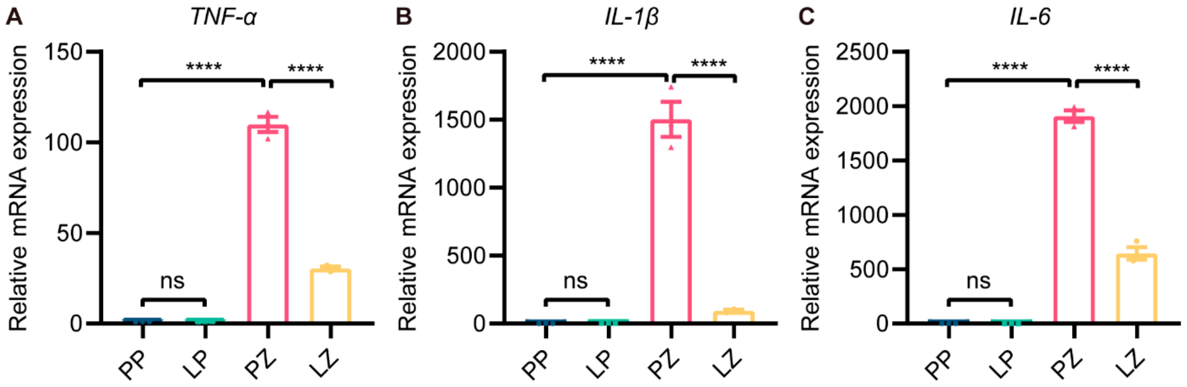

Supplementary Figure S4. Low-dose LPS pretreatment induced a tolerance-like response to subsequent zym challenge in BV2 microglia. The mRNA expression of *TNF-α* **(A)**, *IL-1β* **(B)** and *IL-6* **(C)** was quantified 3 h after the second stimulation. The data are presented as the mean ± SEM; one-way ANOVA, ns indicates no significance, ****$p < 0.0001$.

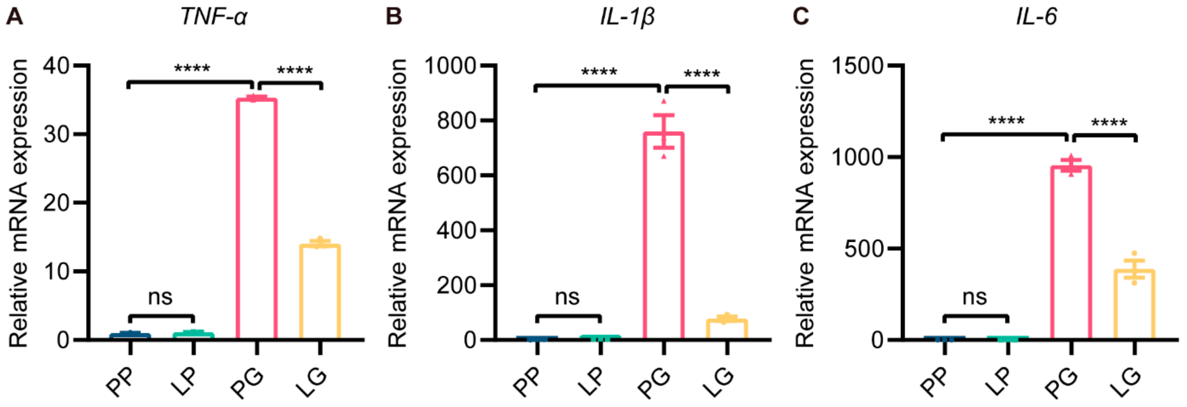

Supplementary Figure S5. Low-dose LPS pretreatment induced a tolerance-like response to subsequent β-Glu challenge in BV2 microglia. The mRNA expression of *TNF-α* **(A)**, *IL-1β* **(B)** and *IL-6* **(C)** was quantified 3 h after the second stimulation. The data are presented as the mean ± SEM; one-way ANOVA, ns indicates no significance, ****$p < 0.0001$.

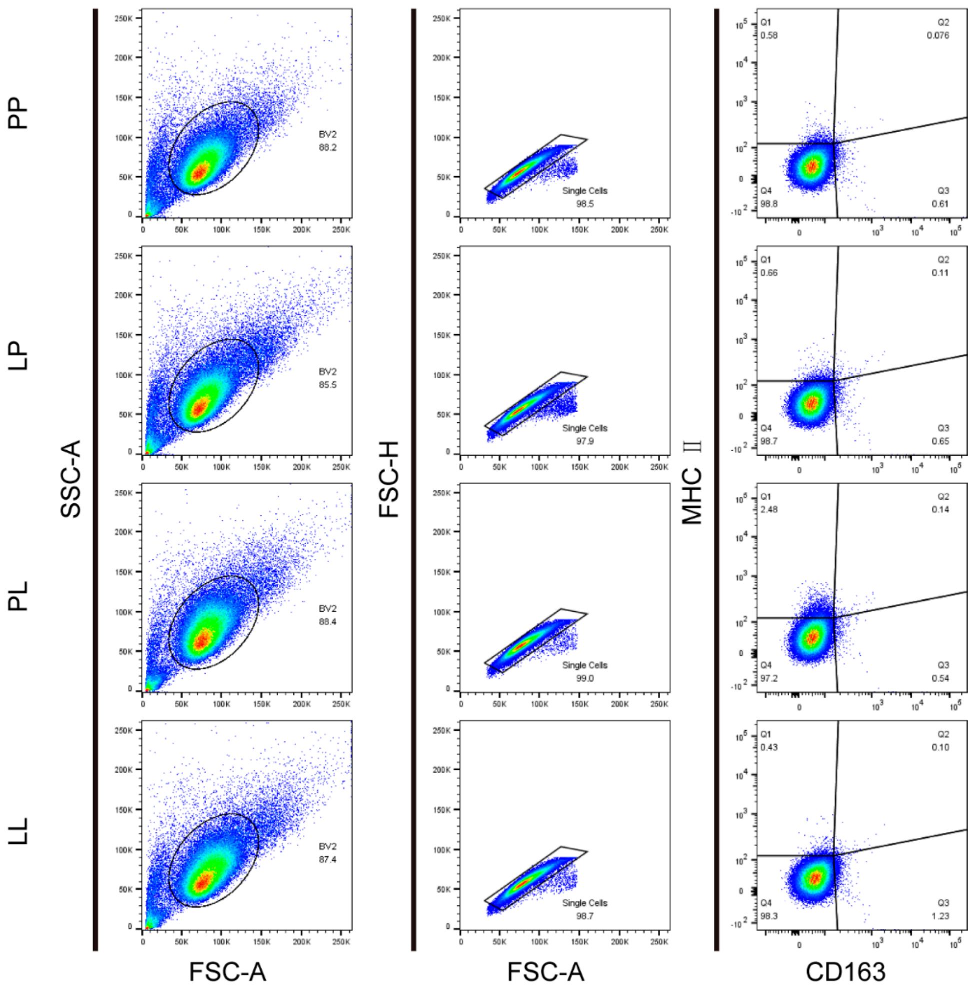

Supplementary Figure S6. Low-dose LPS pretreatment promoted phenotypic transition in BV2 microglia. Representative flow cytometry gating strategy for assessing microglial polarization. MHC II and CD163 were used to identify M1 and M2 microglia, respectively.

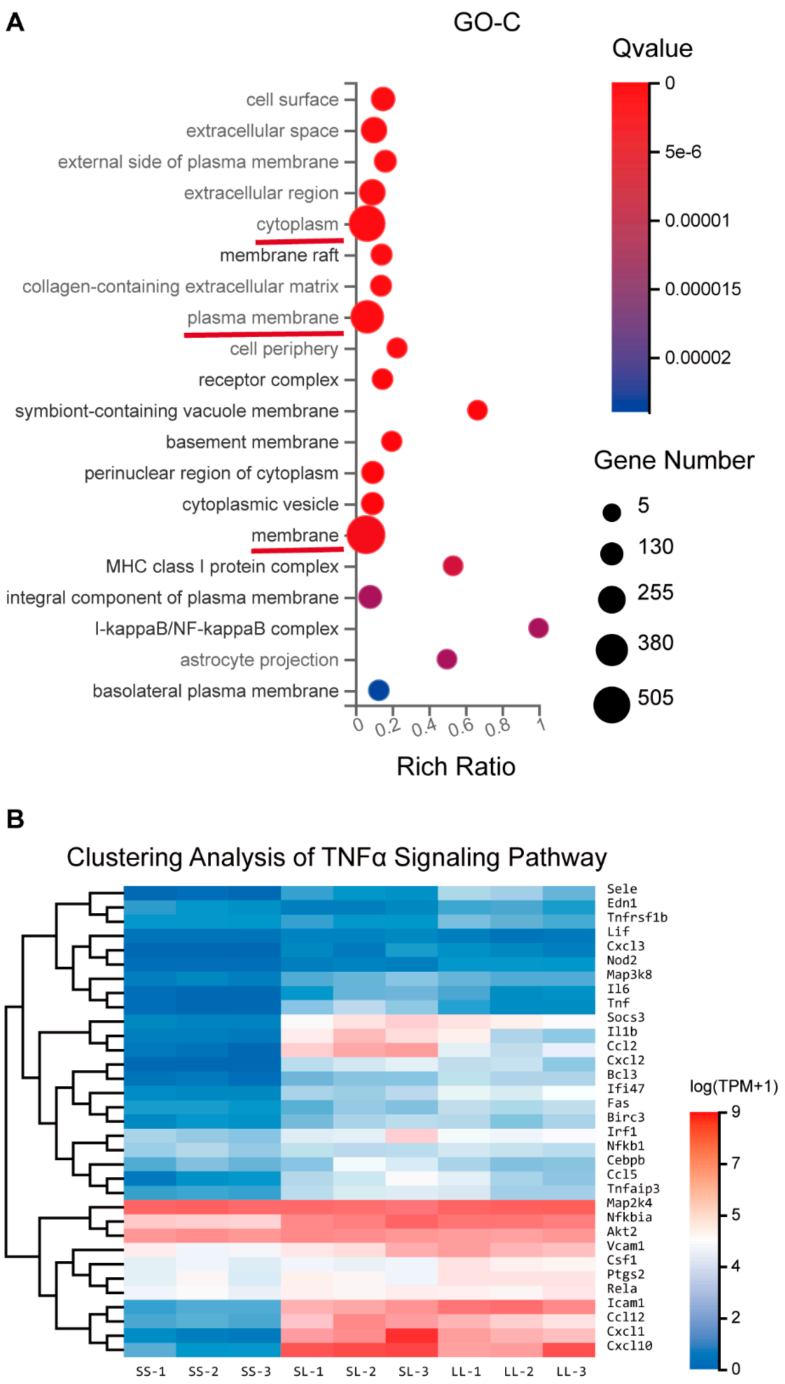

Supplementary Figure S7. Transcriptional profile changes in tolerance-like mice. **(A)** GO-C enrichment analysis of differentially expressed genes between the SL and LL groups. **(B)** Heatmap showing the expression of genes of interest in the SS, SL and LL groups.

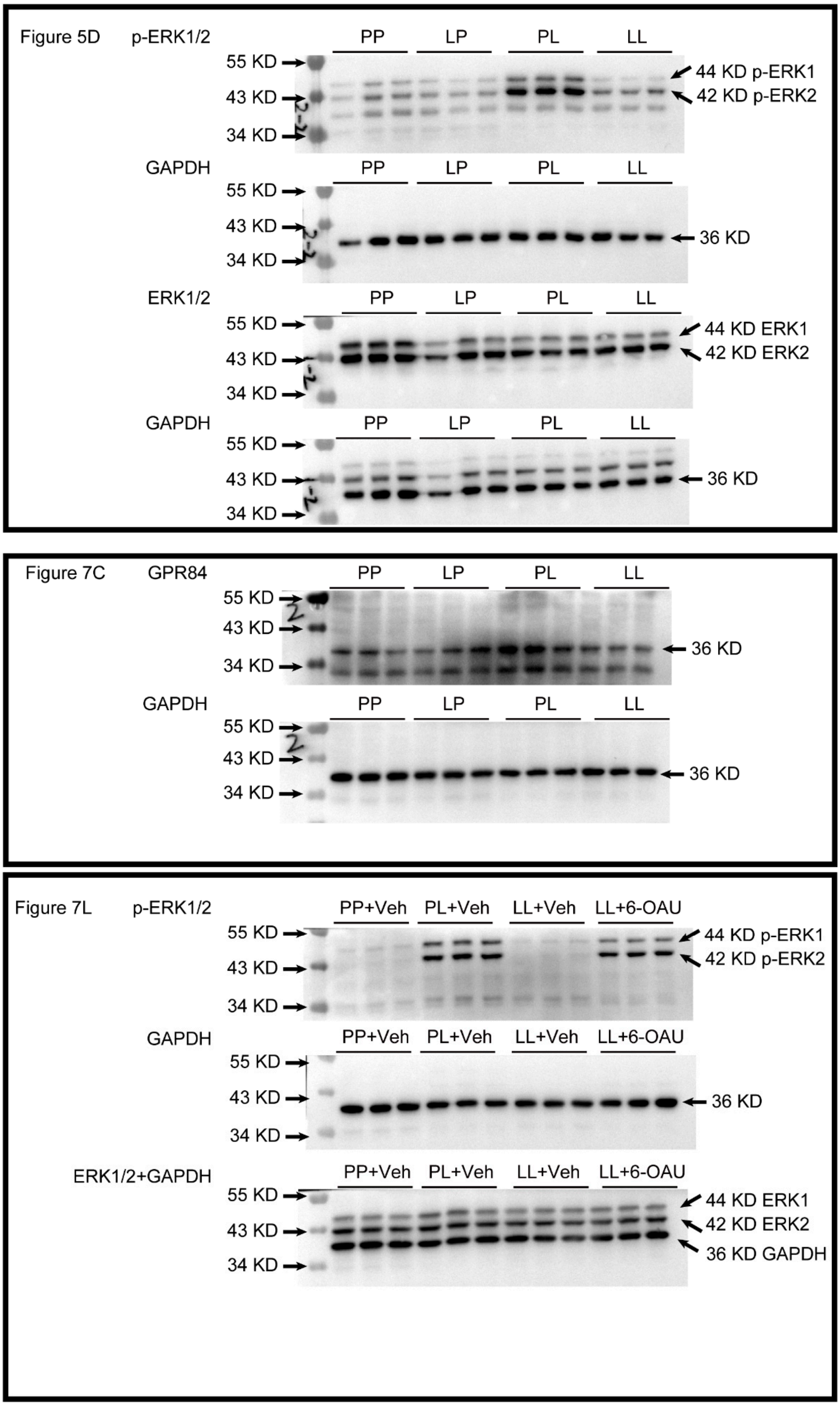

Supplementary Figure S8. Raw images of the western blot in Figure 5D, Figure 7D and Figure 7L.

1.2 Supplementary Tables

Supplementary Table S1. Primary antibody used in WB.

Primary antibody	Dilution	Molecular Weight (kDa)	Host Species	Catalogo	Company
p-ERK1/2	1/1000	42/44	Rabbit	AF1891	Beyotime
ERK1/2	1/1000	42/44	Rabbit	AF1051	Beyotime
GPR84	1/2000	36	Rabbit	bs-15353R	BIOSS
BDNF	1/10000	15	Rabbit	ab108319	Abcom
Synap	1/20000	34	Rabbit	ab32127	Abcom
PSD95	1/2000	95	Goat	ab12093	Abcom
GAPDH	1/2000	36	Rabbit	30202ES40	Yeasen

Supplementary Table S2. Secondary antibody used in WB.

Secondary Antibody	Dilution	Catalogy	Company
Rabbit anti Goat	1/5000	SA00001-4	Proteintech
Goat anti Rabbit	1/5000	ZB-5301	ZSGB-Bio

Supplementary Table S3. Primers used in qPCR.

Gene	Forward	Reverse	Host
TNF-α	GACCCTCACACTCAGATCATCTT	CCTCCACTTGGTGGTTTGCT	Mouse
IL-1β	CTGGTGTGTGACGTTCCCATTA	CCGACAGCACGAGGCTTT	Mouse
IL-6	GCTGGTGACAACCACGGCCT	CTCTCTGAAGGACTCTGGCTTTG	Mouse
IDO1	TGGCGTATGTGTGGAACCG	CTCGCAGTAGGGAACAGCAA	Mouse
CXCL2	CCAACCACCAGGCTACAGG	GCGTCACACTCAAGCTCTG	Mouse
CCL2	GGAATGGGTCCAGACATACATTA	CTACAGAAGTGCTTGAGGTGGT	Mouse
CCL5	TTTGCCTACCTCTCCCTCG	CGACTGCAAGATTGGAGCACT	Mouse
GPR84	GGTGCCAGTTGTCTGCACTT	GCTGAGCCCAAGCACAAAG	Mouse
GAPDH	AATGTGTCCGTCGTGGATCTGA	GATGCCTGCTTCACCACCTTCT	Mouse
