# Supplementary figures and images for: Lipopolysaccharide Preconditioning Restricts Microglial Overactivation and Alleviates Inflammation-Induced Depressive-like Behavior in Mice
Source: Brain Sci. 2023 Mar 25;13(4):549. doi: 10.3390/brainsci13040549 (PMC10137116; doi:10.3390/brainsci13040549)

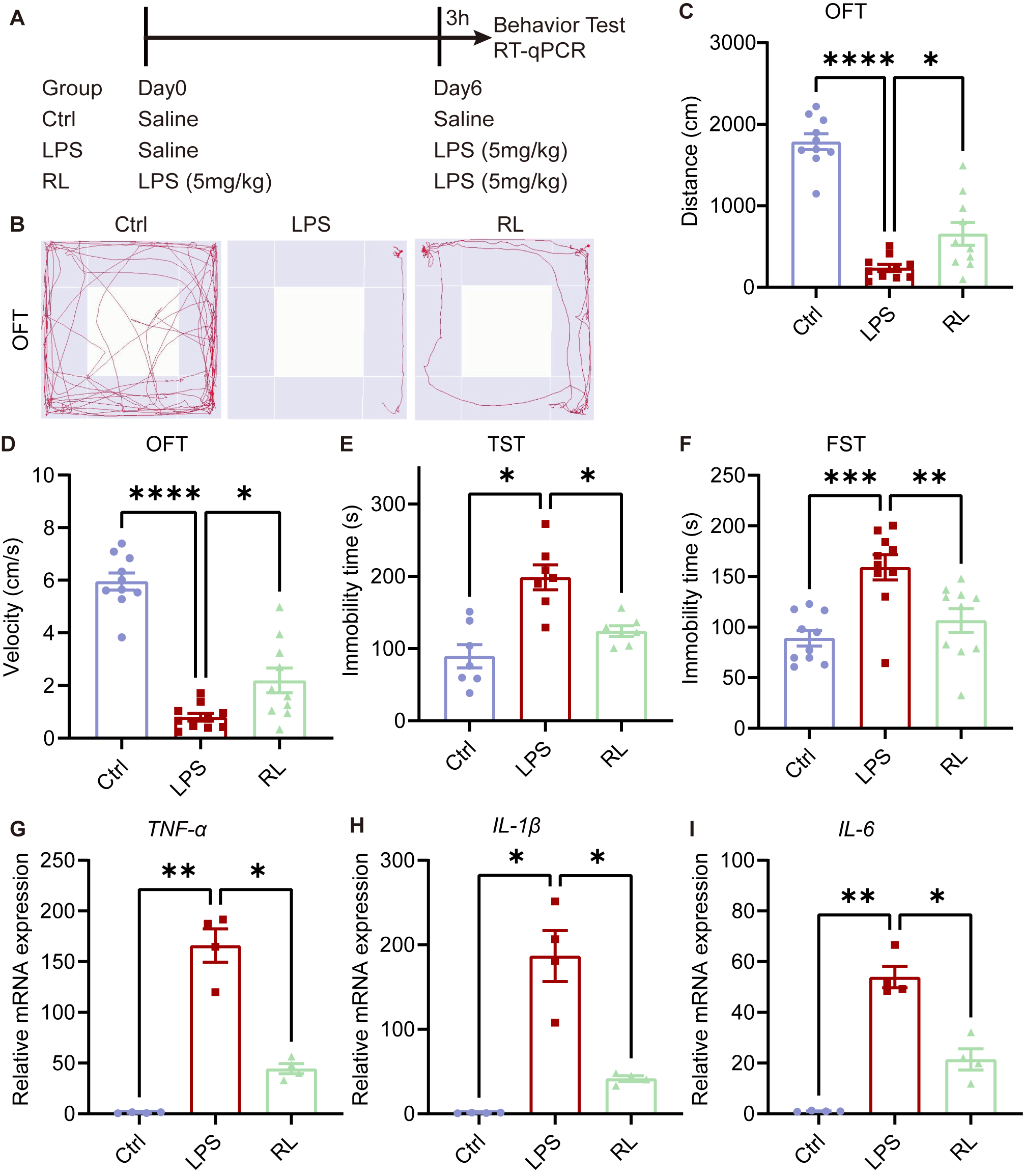

Supplement: Supplementary file 1 [file brainsci-13-00549-s001.zip › SUPPLEMENTARY FIGURE 1.tif]

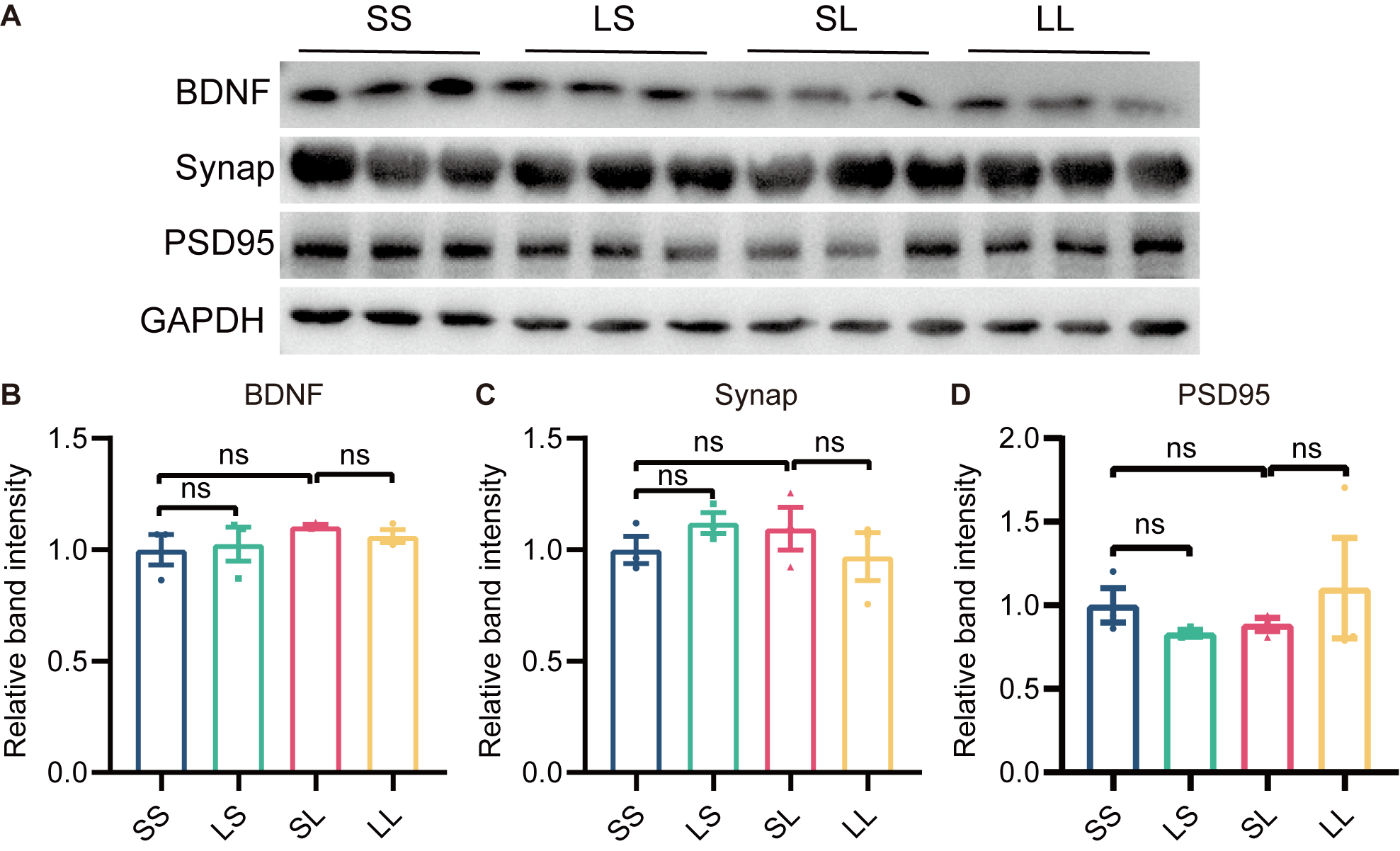

Supplement: Supplementary file 1 [file brainsci-13-00549-s001.zip › SUPPLEMENTARY FIGURE 2.tif]

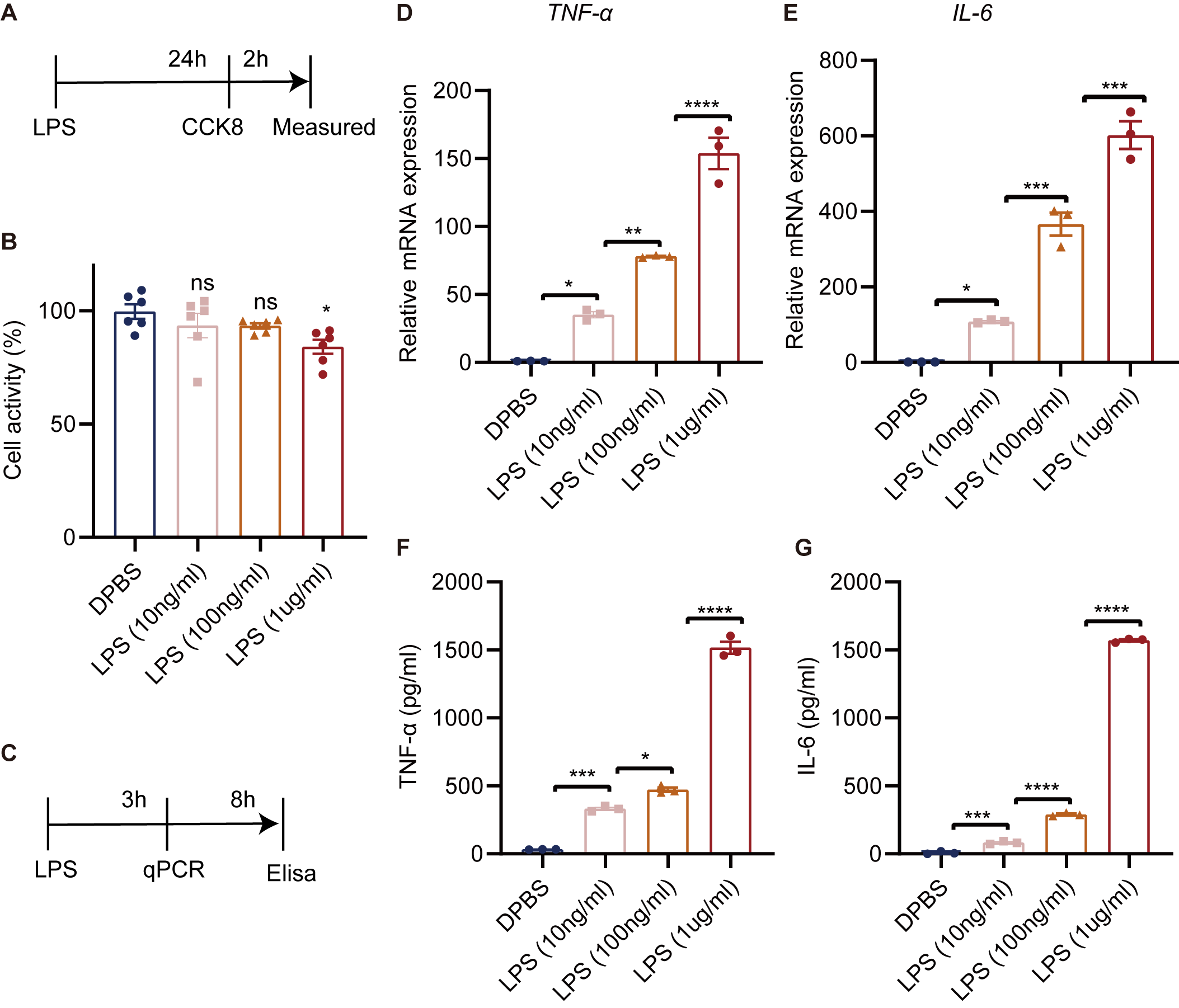

Supplement: Supplementary file 1 [file brainsci-13-00549-s001.zip › SUPPLEMENTARY FIGURE 3.tif]

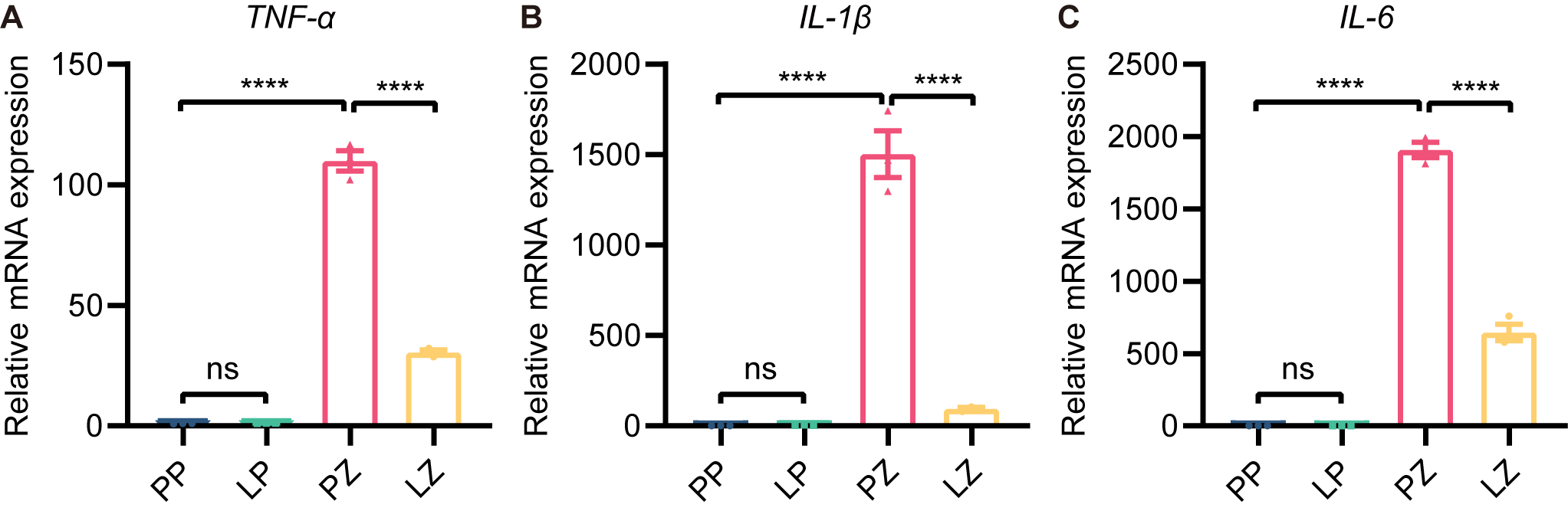

Supplement: Supplementary file 1 [file brainsci-13-00549-s001.zip › SUPPLEMENTARY FIGURE 4.tif]

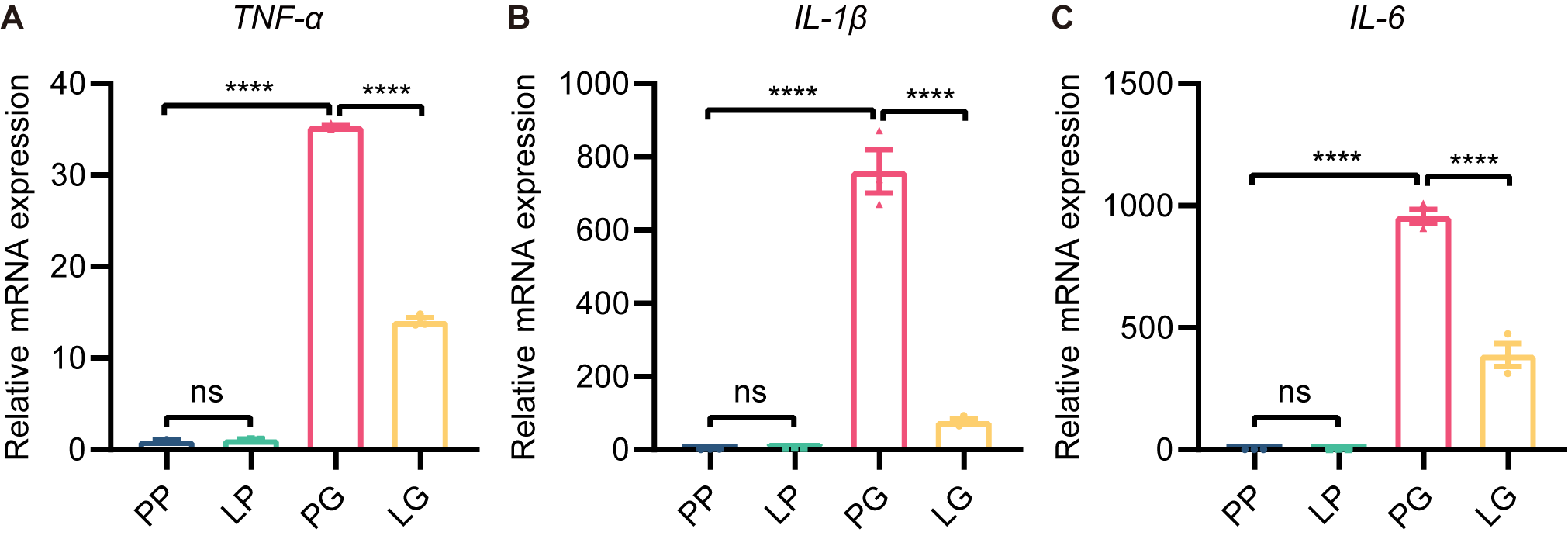

Supplement: Supplementary file 1 [file brainsci-13-00549-s001.zip › SUPPLEMENTARY FIGURE 5.tif]

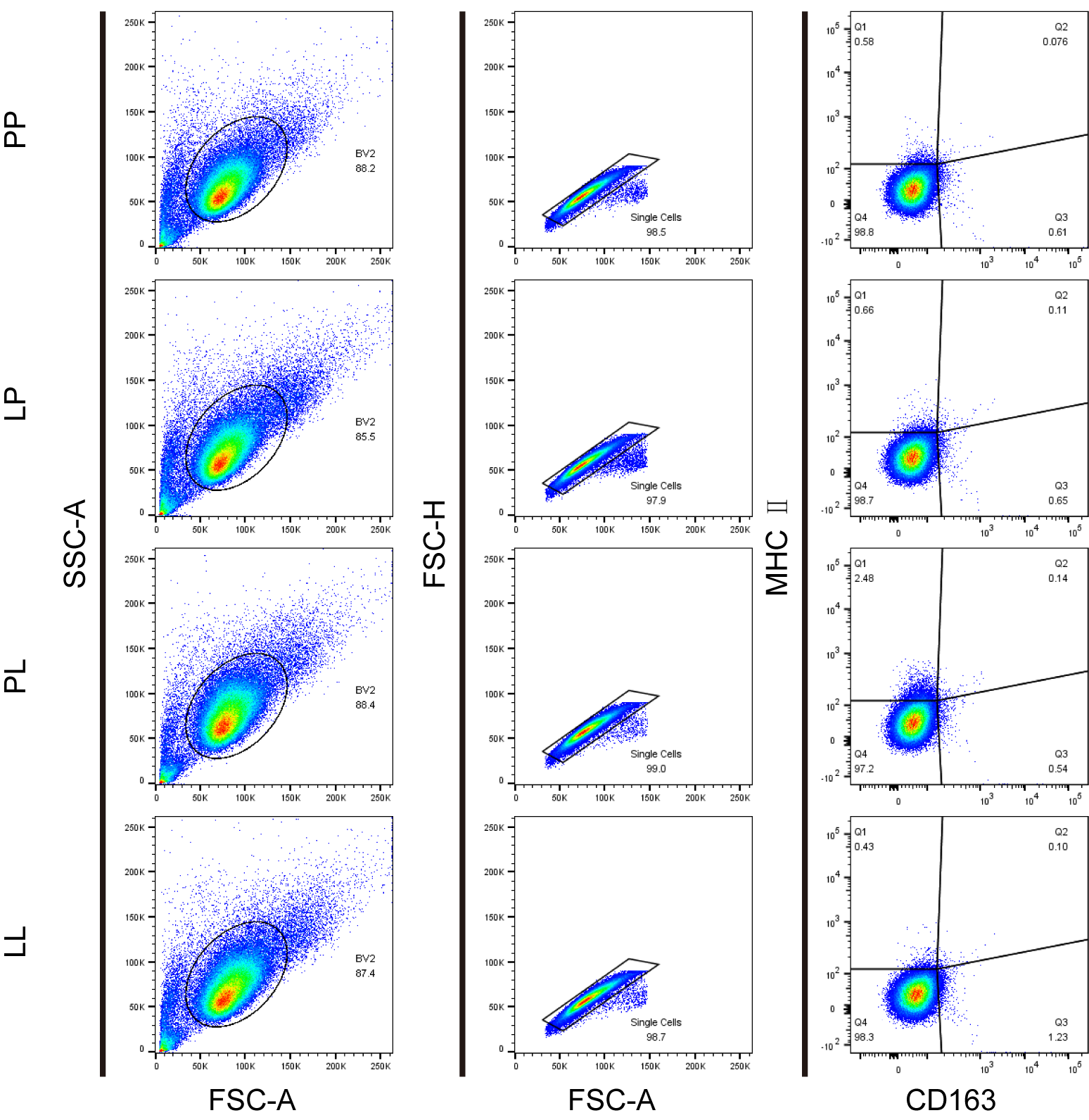

Supplement: Supplementary file 1 [file brainsci-13-00549-s001.zip › SUPPLEMENTARY FIGURE 6.tif]

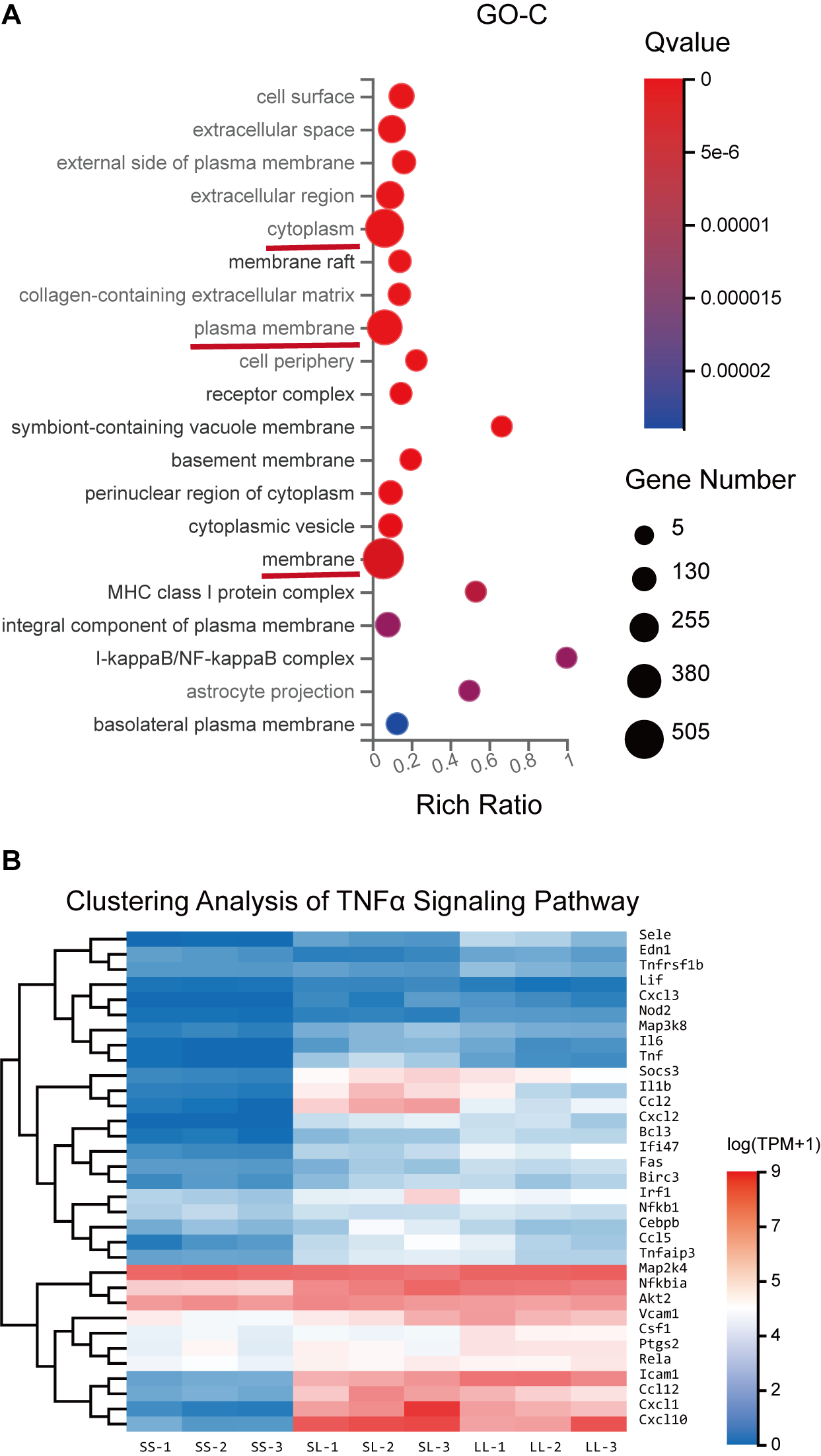

Supplement: Supplementary file 1 [file brainsci-13-00549-s001.zip › SUPPLEMENTARY FIGURE 7.tif]

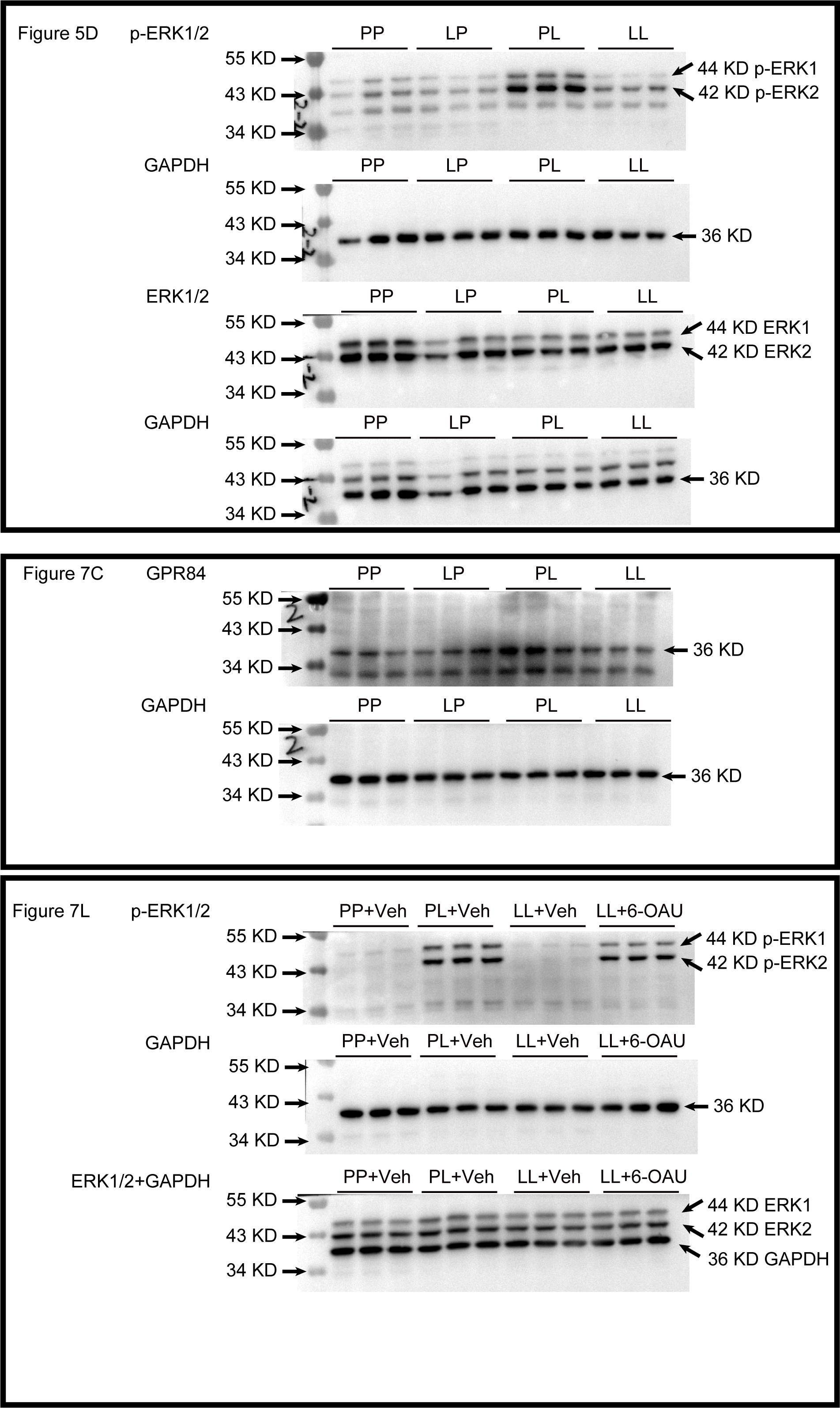

Supplement: Supplementary file 1 [file brainsci-13-00549-s001.zip › SUPPLEMENTARY FIGURE 8.tif]
